# Supplementary material for: A study on Tibetan sheep in the Gansu plague-endemic area and the risk of plague transmission by their vectors
Source: PLoS Negl Trop Dis. 2026 Aug 14;20(8):e0014650. doi: 10.1371/journal.pntd.0014650 (PMC13492742; doi:10.1371/journal.pntd.0014650)
Supplement: Supplementary file 1 [file pntd.0014650.s001.pdf]

PLOS Neglected Tropical Diseases

A Study on Tibetan Sheep in the Gansu Plague-Endemic Area and the Risk of Plague Transmission by Their Vectors

--Manuscript Draft--

**Manuscript Number:**	PNTD-D-26-00605
**Full Title:**	A Study on Tibetan Sheep in the Gansu Plague-Endemic Area and the Risk of Plague Transmission by Their Vectors
**Short Title:**	Tibetan Sheep in Gansu Plague-Endemic Area and Vector-Borne Plague Transmission Risk
**Article Type:**	Research Article
**Keywords:**	Tibetan sheep; plague; transmission risk
**Abstract:**	Abstract:<br>Background: In the Qinghai-Tibet Plateau region, Tibetan sheep can contract plague by licking the carcasses of dead marmots or through flea bites. In recent years, pastoral areas in Gansu Province that are designated as plague foci have introduced Tibetan sheep on a large scale, increasing the risk of plague outbreaks. This study aims to clarify the current status of plague outbreaks among Tibetan sheep in the Himalayan marmot plague foci of Gansu Province and their exposure to vectors, and to systematically assess the risk of plague occurrence and transmission among Tibetan sheep in these foci. Based on these findings, targeted risk control measures will be proposed to provide a scientific basis for decision-making aimed at containing plague outbreaks and safeguarding public health and social stability.<br>Methods: Subei Mongol Autonomous County, one of the Himalayan marmot plague foci in Gansu Province, was selected as the sampling area. Blood, nasal swabs, and cutaneous vector samples were collected from Tibetan sheep across different seasons. Samples were tested using a variety of methods, including serology (IHA), molecular biology (PCR), microbiology (bacterial culture), and flea identification. Combining these results with concurrent plague surveillance data from the sampling area, ArcGIS was used to analyze the distribution of sampling sites and their spatial relationship with animal disease hotspots, and SPSS software was employed to comprehensively evaluate the plague infection status of Tibetan sheep in this endemic area.<br>Results: A total of 10 blood samples tested positive for plague F1 antibodies, with a detection rate of 3.33‰ (10/3033). Antibody titers ranged from 1:16 to 1:128. Spatial analysis revealed that the median distance from plague bacterium detection sites to positive F1 antibody sites during the same period was shorter than the distance to negative sites. Parasites were detected on the body surfaces of 21 Tibetan sheep, with a detection rate of 0.7% (21/3033); 6 cases of Steppalixa ticks and 30 cases of Demodex mites were identified. Laboratory testing revealed that 1,090 nasal swabs, 36 parasite samples, and bacterial cultures were all negative.<br>Conclusion: This study is the first to confirm the presence of plague infection in Tibetan sheep within the plague-endemic areas of Gansu Province. If Tibetan sheep come into contact with infected animals or pathogen-carrying vectors and subsequently enter the trade and distribution channels, it will pose unpredictable public health risks. It is recommended that Tibetan sheep be included in the plague surveillance system, with random sampling of relevant materials; health education and control measures for high-risk populations should be strengthened; and further research on plague in Tibetan sheep should be conducted to provide a theoretical basis for optimizing plague prevention and control strategies.
**Additional Information:**	
**Question**	**Response**
**Government Employee**<br><br>Are you or any of the contributing authors	No - No authors are employees of the U.S. government.

an employee of the United States government?<br><br>Manuscripts authored by one or more US Government employees are not copyrighted, but are licensed under a CC0 Public Domain Dedication, which allows unlimited distribution and reuse of the article for any lawful purpose. This is a legal requirement for US Government employees.<br><br>This will be typeset if the manuscript is accepted for publication.	
**Financial Disclosure**<br><br>Enter a financial disclosure statement that describes the sources of funding for the work included in this submission. Review the submission guidelines for detailed requirements. View published research articles from *PLOS Neglected Tropical Diseases* for specific examples.<br><br>This statement is required for submission and **will appear in the published article** if the submission is accepted. Please make sure it is accurate.<br><br>**Funded studies**<br>Enter a statement with the following details:<br>• Initials of the authors who received each award<br>• Grant numbers awarded to each author<br>• The full name of each funder<br>• URL of each funder website<br>• Did the sponsors or funders play any role in the study design, data collection and analysis, decision to publish, or preparation of the manuscript?<br><br>Did you receive funding for this work?	Yes
Please add funding details.<br>as follow-up to "**Financial Disclosure**<br><br>Enter a financial disclosure statement that	Natural Science Foundations of Gansu Province(24JRRA1157,25JRRA840);Research projects of health industry in Gansu province(GSJKKY2025-15,GSJKKY2025-06)

describes the sources of funding for the work included in this submission. Review the submission guidelines for detailed requirements. View published research articles from *PLOS Neglected Tropical Diseases* for specific examples.<br><br>This statement is required for submission and **will appear in the published article** if the submission is accepted. Please make sure it is accurate.<br><br>**Funded studies**<br>Enter a statement with the following details:<br>• Initials of the authors who received each award<br>• Grant numbers awarded to each author<br>• The full name of each funder<br>• URL of each funder website<br>• Did the sponsors or funders play any role in the study design, data collection and analysis, decision to publish, or preparation of the manuscript?<br><br>Did you receive funding for this work?"	
Please select the country of your main research funder (please select carefully as in some cases this is used in fee calculation).<br>as follow-up to "**Financial Disclosure**<br><br>Enter a financial disclosure statement that describes the sources of funding for the work included in this submission. Review the submission guidelines for detailed requirements. View published research articles from *PLOS Neglected Tropical Diseases* for specific examples.<br><br>This statement is required for submission and **will appear in the published article** if the submission is accepted. Please make sure it is accurate.	CHINA - CN

**Funded studies**<br>Enter a statement with the following details:<br>• Initials of the authors who received each award<br>• Grant numbers awarded to each author<br>• The full name of each funder<br>• URL of each funder website<br>• Did the sponsors or funders play any role in the study design, data collection and analysis, decision to publish, or preparation of the manuscript?<br><br>Did you receive funding for this work?"	
**Competing Interests**<br><br>On behalf of all authors, disclose any competing interests that could be perceived to bias this work.<br><br>This statement will be typeset if the manuscript is accepted for publication.<br><br>Review the instructions link below and PLOS NTDs' competing interests policy to determine what information must be disclosed at submission.	There are no conflicts of interest to declare in this article.
**Data Availability**<br><br>Provide a **Data Availability Statement** in the box below. This statement should detail where the data used in this submission can be accessed. This statement will be typeset if the manuscript is accepted for publication.<br><br>Before publication, authors are required to make all data underlying their findings fully available, without restriction. Review our PLOS Data Policy page for detailed information on this policy. Instructions for writing your Data Availability statement can be accessed via the Instructions link below.	all raw data and analytical methods are fully documented and available for peer review and future research.

Powered by Editorial Manager® and ProduXion Manager® from Aries Systems Corporation

Manuscript

Click here to access/download;Manuscript;A Study on Tibetan Sheep in the Gansu Plague.docx

A Study on Tibetan Sheep in the Gansu Plague-Endemic Area and the Risk of Plague Transmission by Their Vectors

Wenjing An[a,1],Aiwei He[a,1],Guotian Ma[a,1],Hua Chun[d,1],geli Aoer[c,1], Dingsheng Wang[a,1], Ruixun Gan[a],Guorui Wang[a], Jinxiao Xi[a], Hu Wang[b], Chengxin Zhang[c], Yanyan Huang[c], Bayier Caoge[c],Cunshou Zhao[c],Daqin Xu[a,*]

[a]Gansu Provincial Center for Disease Control and Prevention,Lanzhou,Gansu province,China
[b]Jiuquan city Center for Disease Control and Prevention,Jiuquan,Gansu province,China
[c]Subei Mongolian Autonomous Center for Disease Control and Prevention,Subei,Jiuquan,Gansu province,China
[d]Yanchiwan Township Health Center,Subei,Jiuquan,Gansu province,China

Supported by the Natural Science Foundations of Gansu Province(24JRRA1157,25JRRA840);Research projects of health industry in Gansu province(GSJKKY2025-15,GSJKKY2025-06)
Corresponding author:Daqin Xu,Email:317645180@qq.com

Abstract:

Background: In the Qinghai-Tibet Plateau region, Tibetan sheep can contract plague by licking the carcasses of dead marmots or through flea bites. In recent years, pastoral areas in Gansu Province that are designated as plague foci have introduced Tibetan sheep on a large scale, increasing the risk of plague outbreaks. This study aims to clarify the current status of plague outbreaks among Tibetan sheep in the Himalayan marmot plague foci of Gansu Province and their exposure to vectors, and to systematically assess the risk of plague occurrence and transmission among Tibetan sheep in these foci. Based on these findings, targeted risk control measures will be proposed to provide a scientific basis for decision-making aimed at containing plague outbreaks and safeguarding public health and social stability.

Methods: Subei Mongol Autonomous County, one of the Himalayan marmot plague foci in Gansu Province, was selected as the sampling area. Blood, nasal swabs, and cutaneous vector samples were collected from Tibetan sheep across different seasons. Samples were tested using a variety of methods, including serology (IHA), molecular biology (PCR), microbiology (bacterial culture), and flea identification. Combining these results with concurrent plague surveillance data from the sampling area, ArcGIS was used to analyze the distribution of sampling sites and their spatial relationship with animal disease hotspots, and SPSS software was employed to comprehensively evaluate the plague infection status of Tibetan sheep in this endemic area.

Results: A total of 10 blood samples tested positive for plague F1 antibodies, with a detection rate of 3.33‰ (10/3033). Antibody titers ranged from 1:16 to 1:128. Spatial analysis revealed that the median distance from plague bacterium detection sites to positive F1 antibody sites during the same period was shorter than the distance to negative sites. Parasites were detected on the body surfaces of 21 Tibetan sheep, with a detection rate of 0.7% (21/3033); 6 cases of Steppalixa ticks and 30 cases of Demodex mites were identified. Laboratory testing revealed that 1,090 nasal swabs, 36 parasite samples, and bacterial cultures were all negative.

Conclusion: This study is the first to confirm the presence of plague infection in Tibetan sheep within the plague-endemic areas of Gansu Province. If Tibetan sheep come into contact with infected animals or pathogen-carrying vectors and subsequently enter the trade and distribution channels, it will pose unpredictable public health risks. It is recommended that Tibetan sheep be

included in the plague surveillance system, with random sampling of relevant materials; health education and control measures for high-risk populations should be strengthened; and further research on plague in Tibetan sheep should be conducted to provide a theoretical basis for optimizing plague prevention and control strategies.
Keywords: Tibetan sheep; plague; transmission risk

Background

Plague is a severe infectious disease caused by *Yersinia pestis*. As a statutory Category A infectious disease under the Law of the People's Republic of China on the Prevention and Treatment of Infectious Diseases, plague is characterized by extremely high mortality and potential for weaponization as a biological agent. Accordingly, it has long been a major public health priority and a focal concern of the international public health community. As a natural focal disease, the long-term maintenance of plague in the natural environment relies on the synergistic interplay of four core components: the pathogen, vertebrate hosts, arthropod vectors, and suitable habitats. Stable natural plague foci can only be established when all four components are fully satisfied [1]. Among these key components, vertebrate hosts act as critical pathogen reservoirs and amplifiers, and their species composition, population density, and ecological niche directly determine the nature of plague foci and the risk of epidemic outbreaks.

Within the plague transmission cycle, hosts are generally categorized into primary and secondary hosts. Primary hosts represent the pivotal populations responsible for sustaining the enzootic circulation of *Yersinia pestis* in nature, and 14 primary host species have been documented in China's natural plague foci [2]. Secondary hosts (also referred to as incidental hosts) do not contribute to the long-term enzootic maintenance of the pathogen, but can trigger acute epizootic outbreaks following infection and serve as important direct or indirect sources of human infection. To date, 72 secondary host species have been identified across China [2]. In typical plague foci of the Qinghai-Tibet Plateau, primary hosts are predominantly rodent species such as marmots. Notably, Tibetan sheep (*Ovis aries)* have emerged as epidemiologically significant secondary hosts due to their high infection prevalence and documented association with human plague outbreaks, primarily via direct contact with infected or deceased sheep. Consequently, Tibetan sheep have become a core target for local plague surveillance and control interventions.

Surveillance data reveal that between 1958 and 2020, 16 out of 198 laboratory-confirmed human plague cases in Qinghai Province were attributed to direct or indirect transmission from Tibetan sheep [3], highlighting the substantial role of Tibetan sheep in regional plague transmission dynamics. Gansu Province harbors two categories and three subtypes of natural plague foci: the Himalayan marmot plague focus in the alpine steppe of the Qilian Mountains-Altun Mountains, the Himalayan marmot plague focus in the alpine meadow steppe of southern Gansu, and the Alashan ground squirrel plague focus on the Loess Plateau of central Gansu. Among these, the Himalayan marmot plague focus in the alpine steppe of the Qilian

Mountains-Altun Mountains remains epidemiologically active. Since 2000, a total of 10 human plague cases with 8 fatalities have been reported in this focus, corresponding to an 80% case fatality rate [4]. The majority of human infections were linked to direct contact with infected marmots or sheepdogs.

In the Qinghai-Tibet Plateau region, Tibetan sheep are susceptible to plague infection via two main routes: licking carcass bones of infected marmots, or being bitten by fleas that have migrated from marmot carcasses [3]. In recent years, the rapid expansion of animal husbandry has led to a dramatic increase in Tibetan sheep populations within Himalayan marmot plague foci [5], elevating the potential for cross-species pathogen transmission and human exposure risk. This study was designed to characterize the current epidemiological status of plague in Tibetan sheep and their vector exposure profiles in Himalayan marmot foci of Gansu Province, and to systematically evaluate the risk of plague infection and transmission among local Tibetan sheep populations. Based on the empirical findings, targeted risk mitigation strategies will be proposed to provide a scientific evidence base for decision-making, thereby facilitating the prevention and control of plague outbreaks and safeguarding public health and social stability.

Research Methods

Ethics Statement

This study was conducted in strict accordance with the guidelines for animal experimentation and was approved by the Ethics Committee of Gansu Provincial Center for Disease Control and Prevention. All procedures involving animals were performed following the Regulations on the Administration of Laboratory Animals of the People's Republic of China and the Guidelines for the Care and Use of Laboratory Animals. Prior informed consent was obtained from all herders/owners of the Tibetan sheep included in this study. The study protocol was designed to minimize animal suffering, and all sampling procedures were performed by trained veterinary professionals using standard aseptic techniques. No animals were euthanized for the purpose of this study.

Sample Collection

Sampling for this study was conducted during March-May 2024-2025, when animal plague began to spread and marmots emerged from hibernation (hereinafter referred to as“spring sampling”), and during September–November 2024–2025, when the animal plague epidemic subsided and marmots entered hibernation (hereinafter referred to as “autumn sampling”). The sampling site was selected in Suobei Mongol Autonomous County (hereinafter referred to as “Suobei County”), a plague-endemic county with relatively active animal and human plague transmission. Venous blood, nasal swabs, and external parasites were collected from Tibetan sheep within the study area.

Sample Preparation

After collection, centrifuge venous blood at 2000 rpm for 10 minutes to separate the serum. Pipette the supernatant into sample storage tubes. After inactivation in a 56°C water bath for 30 minutes, the serum is ready for subsequent testing. Nasal swabs are collected and stored in bacterial preservation solution for testing. After taxonomic

identification, external parasites are ground in PBS buffer for subsequent testing.

Sample Testing

Using the serum processed as described above, F1 antibody testing was performed in accordance with the operating instructions for the *Yersinia pestis* F1 Antibody Detection Kit and Appendix E of the Indirect Hemagglutination Assay (IHA) for Plague (WS279-2008) in the "Diagnostic Criteria for Plague" [6]. Both nasal swabs and ground samples of external parasites were subjected to molecular biological testing and bacterial culture. For molecular biological testing, nucleic acids were first extracted using a tissue genomic DNA extraction kit, followed by PCR amplification and electrophoresis. The amplification system for each sample consisted of 15 μl of 2× PCR Mix, 0.5 μl of fraF, 0.5 μl of fraR, 0.5 μl of plaF, 0.5 μl of plaR, 1 μl of the sample to be tested, and 12 μl of nucleic acid-free water; the amplification conditions were 5 min at 95°C for pre-denaturation, followed by 30 cycles of 1 min at 95°C, 1 min at 55°C, and 1 min at 72°C, with a final extension of 5 min at 72°C. Bacterial cultures were performed using Hersh medium at 28°C for at least 24 hours, after which colony growth and morphological characteristics were observed. All detection reagents are listed in Table 1.

Statistical Analysis

Data entry and summarization were performed using Excel; statistical analysis was conducted using SPSS 27.0. For data from multiple independent samples, Fisher's exact test and chi-square tests were performed. All statistical tests were two-tailed; $P < 0.05$ was considered statistically significant. Spatial analysis of relevant data was performed using ArcGIS 10.8.

Table 1 Details of Sample Detection Reagents

Test Name	Equipment/Reagent		
	Name	Manufacturer	Batch Number
Plague Indirect Hemagglutination Assay (IHA)	Plague Antibody Detection Kit	Lanzhou Institute of Biological Products Co., Ltd.	20250301
Nucleic Acid Extraction	Tissue Genomic DNA Extraction Kit (CD170-01)	Lanzhou Meibo Biomedical Technology Co., Ltd.	CD17001250521
Polymerase Chain Reaction (PCR)	DL-1000 Marker	Takara Biotechnology Co., Ltd.	A1001B
	2×PCR MIX	Takara Biotechnology Co., Ltd.	A061983A
	GelRed Nucleic Acid Stain	Takara Biotechnology Co., Ltd.	20250314
	5×TBE Buffer	Beijing Solarbio Science & Technology Co., Ltd.	240001001
Bacterial Culture	Herrold's Medium	Beijing Luqiao Technology Co., Ltd.	250116

Results

Sample Collection Results

A total of 3,033 Tibetan sheep were sampled for this study, with 1,329 sampled in the spring and 1,704 in the autumn. The collected samples originated from Nan ning Guo le Village (926 sheep), Kui teng Guo le(185 sheep), and Diao er li ji(202 sheep) in Yan chi wan Township, Subei County; Jin gou(92 sheep) and Shi ban dun(32 sheep) in Shi bao cheng; Ying zui shan(16), Ha shi ha er(4), Yu er hong Pasture (1,102), and Yu er hong Village (474)—totaling 35 sheep herds from 9 villages (Table 2). Among these, 4 herding sites were located at altitudes between 2,500 and 3,000 meters, while the remaining sites were situated between 3,000 and 4,000 meters. A total of 3,033 serum samples, 1,090 nasal swab samples, and 36 Tibetan sheep (22 groups) were collected for examination of external parasites.

Sample Test Results

Results of the indirect hemagglutination test for plague showed that 10 of the 3,033 Tibetan sheep serum samples tested positive for plague F1 antibody, with a positivity rate of 3.3‰ (10/3,033). Among these, the highest seroprevalence of plague antibodies was found in Tibetan sheep from Jin gou, at 10.8‰ (1/92); followed by Yu er hong Pasture, with a seroprevalence of 5.4‰ (6/1102); and Nan ning guo le, with a seroprevalence of 3.2‰ (3/926); no positive serum samples were detected in the remaining villages. Regarding the plague F1 antibody titers in the positive samples, titers ranged from 1:16 to 1:128, including 1 sample at 1:128, 1 at 1:64, 1 at 1:32, and 7 at 1:16. Overall, antibody titers were relatively low and distributed sporadically (Table 3). The 10 positive samples were collected from six different sheep flocks. Among them, five positive samples were detected in the flock of 387 Tibetan sheep belonging to Herder D, which had the highest positive rate of 13.0‰ (5/387); the positive rates for the flocks of the remaining five herders ranged from 2.5‰ to 9.5‰.

Table 2: Number of Tibetan Sheep Samples Collected in Suobei Mongol Autonomous County, Gansu Province

Township	Village	Spring Sampling		Autumn Sampling		Total	
		Number of Households	Households Sampled	Number of Households	Households Sampled	Number of Households	Households Sampled
Yan chi wan	Nan ning guo le	6	290	6	636	12	926
	Kui teng guo le	—	—	3	185	3	185
	Diao er li ji	3	202	—	—	3	202
Shi bao cheng	Jin gou	—	—	1	92	1	92
	Shi ban dun	—	—	2	32	2	32
	Ying zui mountain	—	—	1	16	1	16

	Ha shi ha er	2	4	—	—	2	4
	Yu er hong Ranch	3	819	1	283	4	1102
	Yu er hong	1	14	6	460	7	474
Total		15	1329	20	1704	35	3033

Table 3 Results of F1 Antibody Detection for Plague in Tibetan Sheep Sera

Region		Tested Samples	Positive Samples	Positive Rate	Positive Sample Herder ID	Positive Sample ID	Sample Titer	Positive Sample Season
Township	Village							
Yan chi wan	Nan ning guo le	926	3	3.2‰	A	Y1-74（W）	1：16	Autumn
					B	Y893	1：16	Spring
					C	Y1278	1：16	Autumn
Shi bao cheng	Yu er hong Ranch	1102	6	5.4‰	D	H503	1：32	Spring
					D	H635	1：16	Spring
					D	H670	1：16	Spring
					D	H785	1：16	Spring
					D	H807	1：128	Spring
					E	H918	1：64	Spring
Shi bao cheng	Jin gou	92	1	10.8‰	F	H2540	1：16	Autumn
Yan chi wan	Kui teng guo le	185	0	0‰	—	—	—	—
	Diao er li ji	202	0	0‰	—	—	—	—
Shi bao cheng	Shi ban dun	32	0	0‰	—	—	—	—
	Ying zui mountain	16	0	0‰	—	—	—	—
	Ha shi ha er	4	0	0‰	—	—	—	—
	Yu er hong	474	0	0‰	—	—	—	—
Total		3033	10	3.3‰				

Combined with the results of *Yersinia pestis* detection from the same period in this epidemic area, spatial analysis showed that the median distance from *Yersinia pestis* detection sites to sites with positive *Yersinia pestis* F1 antibody results in Tibetan sheep serum (46,456m) was shorter than the median distance to sites with negative results (61,689m); however, the difference between the two groups was not statistically significant ($U=85$, $Z=-0.09$, $P=0.93$), suggesting that, given the current sample size, the spatial association analysis between seropositive Tibetan sheep and *Yersinia pestis* detection sites has not yet reached statistical significance.

Combing examinations of Tibetan sheep for external parasites yielded a detection rate of 0.70% (21/3033). The 21 affected sheep were from five different flocks, with detection rates as follows: Flock B 0.70% (1/150), Flock C 2.30% (9/400), Herd G: 64.0% (7/11), Herd H: 2.24% (2/89), and Herd I: 4.0% (2/50). The differences in the detection rates of ectoparasites among the different herds were statistically significant (Fisher's Exact Test, $P < 0.001$). A total of 36 ectoparasites were collected, comprising 6 grass ticks, accounting for 16.70% (6/36), and 30 demodex mites, accounting for 83.30% (30/36) (Table 4).

Table 4 Classification and Identification of Ectoparasites on Tibetan Sheep

NO.	Township	Village	Herder ID	Tibetan Sheep ID	Vector Classification Quantity		Sample Collection Season	Total
					Grassland Hard Tick	Chigger Mite		
1	Yan chi wan	Nan ning guo le	G	Y103		2	Spring	2
2			G	Y135		1	Spring	1
3			G	Y152		1	Spring	1
4			G	Y181		1	Spring	1
5			G	Y190		6	Spring	6
6			G	Y191		1	Spring	1
7			G	Y194	1		Spring	1
8			H	Y720	1		Spring	1
9			H	Y830	1		Spring	1
10			B	Y977		2	Spring	2
11		Diao er li ji	I	Y596	1		Spring	1
12			I	Y649	1		Spring	1
13		Nan ning guo le	C	Y1002		2	Autumn	2
14			C	Y1005		1	Autumn	1
15			C	Y1009		1	Autumn	1
16			C	Y1088		2	Autumn	2
17			C	Y1216		1	Autumn	1
18			C	Y1254		3	Autumn	3
19			C	Y1318	1	2	Autumn	3
20			C	Y1338		3	Autumn	3
21			C	Y1374		1	Autumn	1
Total					6	30		36

In this study, 1,090 nasal swab samples and 36 parasites (22 groups) were subjected to both molecular biological testing and bacterial culture. The results showed that no corresponding positive bands were observed in the electrophoresis following PCR amplification, indicating that all samples were negative; furthermore, no colonies characteristic of *Yersinia pestis* were observed in the bacterial cultures, indicating that all samples were negative.

Discussion

As one of China's three major coarse-wooled sheep breeds, the Tibetan sheep is an ancient indigenous breed widely distributed across high-altitude regions of the Qinghai-Tibet Plateau, including Tibet, Qinghai, Gansu, Yunnan, and Sichuan. Leveraging its geographical advantages as a highland interior and vast pasture resources, Qinghai Province has gradually developed into a core production area for Tibetan sheep. However, in recent years, Gansu Province has introduced Tibetan sheep to improve its sheep breed structure and increase production, and the scale of its herd is gradually expanding.

The altitude distribution of the 35 Tibetan sheep grazing sites surveyed in this study confirms the breed's high-altitude adaptability: four grazing sites were located between 2,500 and 3,000 meters, while the remaining 31 sites were all above 3,000 meters, fully demonstrating the widespread distribution of Tibetan sheep in high-altitude regions. It is worth noting that as the primary reservoir host for plague outbreaks in Gansu Province, the Himalayan marmot has a survival altitude range of 2,900–5,500 meters [7]. Although a small number of marmot populations may descend along river valleys to riverbank grasslands at around 2,500 meters or to field ridges at around 2,800 meters, their core distribution area significantly overlaps spatially with the grazing areas of Tibetan sheep. This high degree of ecological niche overlap greatly increases the opportunities for contact between Tibetan sheep grazing areas and Himalayan marmots within the same geographical unit, creating natural conditions for the cross-species transmission of *Yersinia pestis*.

Among the 3,033 blood samples collected from the 35 sheep herds mentioned above, a total of 10 samples tested positive for *Yersinia pestis* F1 antibodies, with a positivity rate of 3.3‰ (10/3,033). Antibody titers ranged from 1:16 to 1:128. This is the first serological confirmation of the prevalence of Tibetan sheep plague in this epidemic focus in Gansu Province. This positivity rate is of the same order of magnitude as the 0.9‰–10.7‰ (average 4.7‰) reported from multi-site surveillance in Qinghai Province [8], suggesting that plague infection in Tibetan sheep in this epidemic focus is not an isolated incident but a stable component of the ecological cycle in the region. Furthermore, by combining the patterns of antibody fluctuation in Tibetan sheep [8], the antibody half-life, and the 2-fold decay rule, it can be roughly estimated that the infection occurred between April 2024 and October 2025, which closely aligns with the marmot activity season (April–October annually). As an introduced species, Tibetan sheep have a relatively short history of grazing in the Jiuquan region of Gansu, yet the positivity rate has already reached 3.3‰. This indicates that once introduced into the epidemic focus, Tibetan sheep can be rapidly exposed to *Yersinia pestis* through direct contact, shared pastures, and vector bites. Although no human cases have been detected to date—likely due to factors such as the short duration of Tibetan sheep rearing, limited human contact, and the distance between marmot habitats and residential areas—this epidemic focus possesses a complete "marmot-Tibetan sheep-human" transmission chain. Without proactive surveillance and an immunological barrier among livestock, activation of this chain would significantly increase the risk of sporadic or even clustered human outbreaks.

Vector organisms play a key role in transmitting pathogens within the plague transmission chain. In this study, a total of 36 parasites were detected on the body surfaces of 21 Tibetan sheep, with a detection rate of 0.7% (21/3033) and a total parasite index of 0.012. The flea index is a key indicator for assessing vector density and transmission risk. Although 0.012 is relatively low, when combined with the 0.7% detection rate, it still suggests a potential risk of vector-borne transmission. It is worth noting that the flea index is influenced by various factors such as season, climate, and host density [9–10]; a single-season cross-sectional survey may not fully reflect vector dynamics. Future research should conduct continuous monitoring across multiple seasons and regions to obtain more reliable vector density data. Regarding the types of parasites detected, although the detection of the grass tick and the leather mite does not indicate they are dominant vectors on the body surface of the Himalayan marmot, they are among its primary parasitic vectors. Furthermore, surveys in natural foci have confirmed that these vectors can carry the pathogen responsible for plague [11]. The detection of these two types of parasites ecologically confirms that vector-host switching does indeed occur between marmots and Tibetan sheep. When marmots die from plague, these parasites detach and re-parasitize the bodies of Tibetan sheep. Although the duration of parasitism may be brief, during plague outbreaks, this transient host switching is sufficient to facilitate interspecies transmission of the pathogen.

Molecular biology and bacterial culture tests of nasal swabs and parasite samples were all negative. Concurrently conducted positive and negative controls yielded corresponding results, indicating that the test results are reliable. Possible reasons for the negative sample results include: low pathogen load in the samples, below the limit of detection, resulting in negative results; studies have shown that different animal species exhibit significant differences in their response to *Yersinia pestis* infection, with some animals potentially presenting as subclinical infections or transient carriers [12]. If the sampling timing did not cover the peak period of bacterial shedding, it would be difficult to detect the pathogen. However, in conjunction with the results of the serological survey conducted concurrently with this study (3.3‰), it can be inferred that Tibetan sheep do indeed have a history of exposure to *Yersinia pestis*; however, pathogen evidence may not have been detected due to rapid clearance of the pathogen, inappropriate sampling timing, or limitations in the sensitivity of the detection method. This coexistence of serological positivity and negative pathogen detection is not uncommon in the surveillance of naturally occurring zoonotic diseases, suggesting that Tibetan sheep may primarily exhibit a “rapid clearance after infection” host pattern rather than a state of long-term carriage. Tibetan sheep may serve as “terminal hosts” or “sentinel animals” rather than “reservoir hosts.” Although they rapidly clear the pathogen after infection, they retain antibodies, which in turn confirms the activity of the epidemic focus; however, they themselves may not contribute to maintaining the focus. This distinction is crucial for the formulation of control strategies.

Conclusion

In summary, there is a risk of plague infection and transmission among Tibetan sheep

and their vectors in the natural plague foci of the Himalayan marmot in Gansu Province. This study proposes the following targeted control measures: It is recommended that Tibetan sheep be included in the plague host surveillance system, with a focus on sampling and monitoring during plague outbreaks in marmots in endemic areas. Random sampling should be conducted on Tibetan sheep serum in pastoral areas, as well as at cattle and sheep trading markets and related biological materials. Serological and molecular biological methods should be used to detect plague F1 antibodies in the samples; Strengthen health education and behavioral interventions for high-risk groups (herders, sheep traders, and slaughterhouse workers) to enhance their awareness of and capacity for protection, thereby safeguarding their health. Concurrently, in-depth basic research on Tibetan sheep in plague-endemic areas should be conducted, exploring aspects such as infection mechanisms to provide a theoretical basis for optimizing plague prevention and control strategies.

References

[1] Wang X, Jing HQ, Kan B. Long and arduous road: prevalence and prevention of plague in modern society. *Chinese Journal of Tropical Medicine*, 2024, 24(1):22-27+48. DOI:10.13604/j.cnki.46-1064/r.2024.01.05.

[2] Qin CY, Xu L, Zhang RZ, et al. Typing of natural plague foci in China V: biological characteristics of plague hosts. *Chinese Journal of Epidemiology*, 2012, 33(7):692-697.

[3] Xiong HM, Jin J, Xin YQ, et al. Phylogenetic relationship and epidemiological characteristics of *Yersinia pestis* isolated from Tibetan sheep on the Qinghai Plateau. *Chinese Journal of Disease Control & Prevention*, 2022, 26(12):1473-1478. DOI:10.16462/j.cnki.zhjbkz.2022.12.019.

[4] Cong XB, Ju C. Human Plague in China. Beijing: People's Medical Publishing House, 2018:92-104.

[5] Cai LQ, Kan GQ, Ma L, et al. Current status of animal husbandry development and paths for cultivating superior and characteristic industries in Subei County. *Gansu Animal Husbandry and Veterinary Medicine*, 2024, 54(5):128-132. DOI:10.15979/j.cnki.cn62-1064/s.2024.05.002.

[6] Ministry of Health of the People's Republic of China. Diagnostic criteria for plague (WS279-2008). Beijing: Standards Press of China, 2008:10-11.

[7] Bao ZQ, Li C, Guo C, et al. Convergent evolution of Himalayan marmot with some high-altitude animals through ND3 protein. *Animals (Basel)*, 2021, 11(2):251.

[8] Yu DZ. Animal Epidemiology of Plague. Beijing: Science Press, 2009:263-271.

[9] Li ZL, Wang CG, Ma LM. Relationship between density of *Spermophilus dauricus*, meteorological factors and flea index. *Chinese Journal of Vector Biology and Control*, 1993, (4):282-283.

[10] Wang DD, Xu DQ, Ge PF, Xi JX, et al. Relationship between the number of parasitic fleas on *Spermophilus alashanicus*, host abundance and meteorological factors. *Chinese Journal of Endemiology*, 2018, 37(12):965-968.

[11] Li HL, Wei YW, Li C, et al. Cluster analysis of medical gamasid mites in Qinghai Province. *Chinese Journal of Zoonoses*, 2014, 30(1):67-73.

[12] Chen M, Shi LY. Research progress on susceptibility, resistance and mechanisms of plague-infected animals to *Yersinia pestis*. *Chinese Journal of Endemiology*, 2023, 42(7):593-597.
