# Supplementary material for: A study on Tibetan sheep in the Gansu plague-endemic area and the risk of plague transmission by their vectors
Source: PLoS Negl Trop Dis. 2026 Aug 14;20(8):e0014650. doi: 10.1371/journal.pntd.0014650 (PMC13492742; doi:10.1371/journal.pntd.0014650)
Supplement: Supplementary file 2 [file pntd.0014650.s003.pdf]

PLOS Neglected Tropical Diseases

A Study on Tibetan Sheep in the Gansu Plague-Endemic Area and the Risk of Plague Transmission by Their Vectors

--Manuscript Draft--

**Manuscript Number:**	PNTD-D-26-00605R1
**Full Title:**	A Study on Tibetan Sheep in the Gansu Plague-Endemic Area and the Risk of Plague Transmission by Their Vectors
**Short Title:**	Tibetan Sheep in Gansu Plague-Endemic Area and Vector-Borne Plague Transmission Risk
**Article Type:**	Research Article
**Keywords:**	Tibetan sheep; Plague; transmission risk
**Abstract:**	Abstract:<br>Background: In the Qinghai-Tibet Plateau region, Tibetan sheep can contract plague by licking the carcasses of dead marmots or through flea bites. In recent years, pastoral areas in Gansu Province that are designated as plague foci have introduced Tibetan sheep on a large scale, increasing the risk of plague outbreaks. This study aims to clarify the current status of plague outbreaks among Tibetan sheep in the Himalayan marmot plague foci of Gansu Province and their exposure to vectors, and to systematically assess the risk of plague occurrence and transmission among Tibetan sheep in these foci. Based on these findings, targeted risk control measures will be proposed to provide a scientific basis for decision-making aimed at containing plague outbreaks and safeguarding public health and social stability.<br>Methods: Subei Mongol Autonomous County, one of the Himalayan marmot plague foci in Gansu Province, was selected as the sampling area. Blood, nasal swabs, and cutaneous vector samples were collected from Tibetan sheep across different seasons. Samples were tested using a variety of methods, including serology (IHA), molecular biology (PCR), microbiology (bacterial culture), and flea identification. Combining these results with concurrent plague surveillance data from the sampling area, ArcGIS was used to analyze the distribution of sampling sites and their spatial relationship with animal disease hotspots, and SPSS software was employed to comprehensively evaluate the plague infection status of Tibetan sheep in this endemic area.<br>Results: A total of 10 blood samples tested positive for plague F1 antibodies, with a detection rate of 3.33‰ (10/3033). Antibody titers ranged from 1:16 to 1:128. Spatial analysis revealed that the median distance from plague bacterium detection sites to positive F1 antibody sites during the same period was shorter than the distance to negative sites. Parasites were detected on the body surfaces of 21 Tibetan sheep, with a detection rate of 0.7% (21/3033); 6 cases of Steppalixa ticks and 30 cases of Demodex mites were identified. Laboratory testing revealed that 1,090 nasal swabs, 36 parasite samples, and bacterial cultures were all negative.<br>Conclusion: This study is the first to confirm the presence of plague infection in Tibetan sheep within the plague-endemic areas of Gansu Province. If Tibetan sheep come into contact with infected animals or pathogen-carrying vectors and subsequently enter the trade and distribution channels, it will pose unpredictable public health risks. It is recommended that Tibetan sheep be included in the plague surveillance system, with random sampling of relevant materials; health education and control measures for high-risk populations should be strengthened; and further research on plague in Tibetan sheep should be conducted to provide a theoretical basis for optimizing plague prevention and control strategies.
**Additional Information:**	
**Question**	**Response**
**Government Employee**<br><br>Are you or any of the contributing authors	No - No authors are employees of the U.S. government.

an employee of the United States government?<br><br>Manuscripts authored by one or more US Government employees are not copyrighted, but are licensed under a CC0 Public Domain Dedication, which allows unlimited distribution and reuse of the article for any lawful purpose. This is a legal requirement for US Government employees.<br><br>This will be typeset if the manuscript is accepted for publication.	
**Financial Disclosure**<br><br>Enter a financial disclosure statement that describes the sources of funding for the work included in this submission. Review the submission guidelines for detailed requirements. View published research articles from *PLOS Neglected Tropical Diseases* for specific examples.<br><br>This statement is required for submission and **will appear in the published article** if the submission is accepted. Please make sure it is accurate.<br><br>**Funded studies**<br>Enter a statement with the following details:<br>• Initials of the authors who received each award<br>• Grant numbers awarded to each author<br>• The full name of each funder<br>• URL of each funder website<br>• Did the sponsors or funders play any role in the study design, data collection and analysis, decision to publish, or preparation of the manuscript?<br><br>Did you receive funding for this work?	Yes
Please add funding details.<br>as follow-up to "**Financial Disclosure**<br><br>Enter a financial disclosure statement that	This work was supported by the Natural Science Foundations of Gansu Province (24JRRA1157 to Dingsheng Wang and 25JRRA840 to Daqin Xu) and the Research Projects of Health Industry in Gansu Province (GSJKKY2025-15 to Daqin Xu and GSJKKY2025-06 to Aiwei He). The funders had no role in study design, data collection

describes the sources of funding for the work included in this submission. Review the submission guidelines for detailed requirements. View published research articles from *PLOS Neglected Tropical Diseases* for specific examples.<br><br>This statement is required for submission and **will appear in the published article** if the submission is accepted. Please make sure it is accurate.<br><br>**Funded studies**<br>Enter a statement with the following details:<br>• Initials of the authors who received each award<br>• Grant numbers awarded to each author<br>• The full name of each funder<br>• URL of each funder website<br>• Did the sponsors or funders play any role in the study design, data collection and analysis, decision to publish, or preparation of the manuscript?<br><br>Did you receive funding for this work?"	and analysis, decision to publish, or preparation of the manuscript. No authors received a salary from any of the funders.
Please select the country of your main research funder (please select carefully as in some cases this is used in fee calculation).<br>as follow-up to "**Financial Disclosure**<br><br>Enter a financial disclosure statement that describes the sources of funding for the work included in this submission. Review the submission guidelines for detailed requirements. View published research articles from *PLOS Neglected Tropical Diseases* for specific examples.<br><br>This statement is required for submission and **will appear in the published article** if the submission is accepted. Please make sure it is accurate.	CHINA - CN

**Funded studies**<br>Enter a statement with the following details:<br>• Initials of the authors who received each award<br>• Grant numbers awarded to each author<br>• The full name of each funder<br>• URL of each funder website<br>• Did the sponsors or funders play any role in the study design, data collection and analysis, decision to publish, or preparation of the manuscript?<br><br>Did you receive funding for this work?"	
**Competing Interests**<br><br>On behalf of all authors, disclose any competing interests that could be perceived to bias this work.<br><br>This statement will be typeset if the manuscript is accepted for publication.<br><br>Review the instructions link below and PLOS NTDs' competing interests policy to determine what information must be disclosed at submission.	The authors have declared that no competing interests exist.
**Data Availability**<br><br>Before publication, Authors are required to make fully available and without restriction all data underlying their findings. Sharing the data underlying your findings increases the visibility, credibility, and impact of your work, while supporting transparency and future scientific discovery. Please see our PLOS Data Policy page for detailed information on this policy.<br><br>A **Data Availability Statement**, detailing where the data can be accessed, is required at first submission. Insert your Data Availability Statement in the box below.	all raw data and analytical methods are fully documented and available for peer review and future research.

Please see the data reporting section of our submission guidelines for instructions on what you need to include in your Data Availability Statement. Examples of acceptable Data Availability Statements are included here for reference:<br>1. The minimal data set [and accompanying code, where generated] is available at [repository name] via [DOI hyperlink]<br>2. No data was generated by this study. The following existing data sources were used: [dataset name] from [dataset location] available via [web address or DOI hyperlink].<br>3. For Study Protocols: No datasets were generated or analysed during the current study. All relevant data from this study will be made available upon study completion.<br><br>*PLOS allows rare exemptions to address legal and ethical concerns. If you have legal or ethical restrictions, please use the box below to detail these in full sentences for the Journal team to consider.*<br><br>This statement will be typeset if the manuscript is accepted for publication.	

Manuscript Click here to access/download;Manuscript;Manuscript.docx

A Study on Tibetan Sheep in the Gansu Plague-Endemic Area and the Risk of Plague Transmission by Their Vectors

Wenjing An[a,1],Aiwei He[a,1],Guotian Ma[a,1],Hua Chun[d,1],geli Aoer[c,1], Dingsheng Wang[a,1], Ruixun Gan[a],Guorui Wang[a], Jinxiao Xi[a], Hu Wang[b], Chengxin Zhang[c], Yanyan Huang[c], Bayier Caoge[c],Cunshou Zhao[c],Daqin Xu[a,*]

[a]Gansu Provincial Center for Disease Control and Prevention,Lanzhou,Gansu province,China
[b]Jiuquan city Center for Disease Control and Prevention,Jiuquan,Gansu province,China
[c]Subei Mongolian Autonomous Center for Disease Control and Prevention,Subei,Jiuquan,Gansu province,China
[d]Yanchiwan Township Health Center,Subei,Jiuquan,Gansu province,China

This work was supported by the Natural Science Foundations of Gansu Province (24JRRA1157 to Dingsheng Wang and 25JRRA840 to Daqin Xu) and the Research Projects of Health Industry in Gansu Province (GSJKKY2025-15 to Daqin Xu and GSJKKY2025-06 to Aiwei He). The funders had no role in study design, data collection and analysis, decision to publish, or preparation of the manuscript. No authors received a salary from any of the funders.
Corresponding author:Daqin Xu,Email:317645180@qq.com

Abstract

Background: In the Qinghai-Tibet Plateau region, Tibetan sheep can contract plague by licking the carcasses of dead marmots or through flea bites. In recent years, pastoral areas in Gansu Province that are designated as plague foci have introduced Tibetan sheep on a large scale, increasing the risk of plague outbreaks. This study aims to clarify the current status of plague outbreaks among Tibetan sheep in the Himalayan marmot plague foci of Gansu Province and their exposure to vectors, and to systematically assess the risk of plague occurrence and transmission among Tibetan sheep in these foci. Based on these findings, targeted risk control measures will be proposed to provide a scientific basis for decision-making aimed at containing plague outbreaks and safeguarding public health and social stability.
Methods: Subei Mongol Autonomous County, one of the Himalayan marmot plague foci in Gansu Province, was selected as the sampling area. Blood, nasal swabs, and cutaneous vector samples were collected from Tibetan sheep across different seasons. Samples were tested using a variety of methods, including serology (IHA), molecular biology (PCR), microbiology (bacterial culture), and flea identification. Combining these results with concurrent plague surveillance data from the sampling area, ArcGIS was used to analyze the distribution of sampling sites and their spatial relationship with animal disease hotspots, and SPSS software was employed to comprehensively evaluate the plague infection status of Tibetan sheep in this endemic area.
Results: A total of 3,033 Tibetan sheep from 35 herds across 9 villages were sampled.10 blood samples tested positive for plague F1 antibodies, with a detection rate of 3.33‰ (10/3033). Antibody titers ranged from 1:16 to 1:128. Spatial analysis revealed that the median distance from plague bacterium detection sites to positive F1 antibody sites during the same period was shorter than the distance to negative sites. Ectoparasites were detected on the body surfaces of 21 Tibetan sheep, with a detection rate of 0.7% (21/3033); 6 hard ticks (Ixodidae) and 30 mites (Acari) were identified. Laboratory testing revealed that 1,090 nasal swabs, 36 parasite samples, and bacterial cultures were all negative.
Conclusion: This study is the first to confirm the presence of plague infection in Tibetan sheep

within the plague-endemic areas of Gansu Province. If Tibetan sheep come into contact with infected animals or pathogen-carrying vectors and subsequently enter the trade and distribution channels, it will pose unpredictable public health risks. It is recommended that Tibetan sheep be included in the plague surveillance system, with random sampling of relevant materials; health education and control measures for high-risk populations should be strengthened; and further research on plague in Tibetan sheep should be conducted to provide a theoretical basis for optimizing plague prevention and control strategies.
Keywords: Tibetan sheep; plague; transmission risk

Author Summary

Plague remains endemic in Himalayan marmot foci of the Qinghai-Tibet Plateau. While Tibetan sheep are known to be susceptible to Yersinia pestis through contact with infected marmots or flea bites,their specific infection status within Gansu Province's plague foci had not been systematically evaluated despite rapid expansion of sheep husbandry.This study presents the first confirmed evidence of plague infection in Tibetan sheep within Gansu, identifying F1 antibodies in 10 of 3,033 sampled animals.Spatial analysis revealed that antibody-positive sheep were located significantly closer to active plague detection sites than negative animals, suggesting ongoing environmental exposure.Although ectoparasite detection was low (0.7%) and all nasal swabs and cultures were negative,seropositive animals indicate that Tibetan sheep are actively exposed to plague in these natural foci.Given the potential for infected sheep to enter trade networks,posing unpredictable public health risks, this research underscores the urgent need to incorporate Tibetan sheep into routine plague surveillance and strengthen health education for high-risk pastoral communities. These findings provide a critical basis for optimizing plague prevention strategies and safeguarding public health in expanding pastoral areas.

Introduction

Plague is a severe infectious disease caused by *Yersinia pestis*.As a statutory Category A infectious disease under the Law of the People's Republic of China on the Prevention and Treatment of Infectious Diseases.The disease is characterized by acute onset, severe clinical course,strong transmissibility,and high case-fatality rate.Due to its high lethality,plague remains one of the priority targets for global public health security prevention and control[1].As a natural focal disease,the long-term maintenance of plague in the natural environment relies on the synergistic interplay of four core components:the pathogen,vertebrate hosts,arthropod vectors,and suitable habitats[2].Stable natural plague foci can only be established when all four components are fully satisfied.Among these key components,vertebrate hosts act as critical pathogen reservoirs and amplifiers,and their species composition, population density, and ecological niche directly determine the nature of plague foci and the risk of epidemic outbreaks.

Within the plague transmission cycle,hosts are generally categorized into primary and secondary hosts.Primary hosts represent the pivotal populations responsible for sustaining the enzootic circulation of *Yersinia pestis* in nature, and 14 primary host

species have been documented in China's natural plague foci [3].Secondary hosts (also referred to as incidental hosts) do not contribute to the long-term enzootic maintenance of the pathogen,but can trigger acute epizootic outbreaks following infection and serve as important direct or indirect sources of human infection[3].To date,72 secondary host species have been identified across China.In typical plague foci of the Qinghai-Tibet Plateau, primary hosts are predominantly rodent species such as marmots.Notably,Tibetan sheep (*Ovis aries)* have emerged as epidemiologically significant secondary hosts due to their high infection prevalence and documented association with human plague outbreaks, primarily via direct contact with infected or deceased sheep.Consequently,Tibetan sheep have become a core target for local plague surveillance and control interventions.

Surveillance data reveal that between 1958 and 2020,16 of 198 laboratory-confirmed human plague cases in Qinghai Province were attributed to direct or indirect transmission from Tibetan sheep[4],and fourteen strains of *Yersinia pestis* were isolated from Tibetan sheep[5].Furthermore,human plague outbreaks linked to Tibetan sheep were documented in Longzi and Damxung,Xizang,in 1992 and 1994, respectively[6,7],underscoring the substantial role of Tibetan sheep in regional plague transmission dynamics.Gansu Province harbors two categories and three subtypes of natural plague foci:the Himalayan marmot plague focus in the alpine steppe of the Qilian Mountains-Altun Mountains,the Himalayan marmot plague focus in the alpine meadow steppe of southern Gansu,and the Alashan ground squirrel plague focus on the Loess Plateau of central Gansu.Among these,the Himalayan marmot plague focus in the alpine steppe of the Qilian Mountains-Altun Mountains remains epidemiologically active.Since 2000, a total of 10 human plague cases with 8 fatalities have been reported in this focus,corresponding to an 80% case fatality rate [8].The majority of human infections were linked to direct contact with infected marmots or sheepdogs.

In the Qinghai-Tibet Plateau region,Tibetan sheep are susceptible to plague infection via two main routes: licking carcass bones of infected marmots,or being bitten by fleas that have migrated from marmot carcasses [4].In recent years, sheep populations have expanded substantially in the Himalayan marmot plague foci of Gansu Province. The sheep inventory in Subei County increased from 337,860 head in 2020 to 414,304 head in 2022[9]. According to field surveys, Tibetan sheep, as a confirmed secondary host of plague, have also increased in number through introduction.The role of sheep in plague ecology extends beyond contemporary transmission dynamics.Recent paleogenomic evidence has identified ancient *Y. pestis* lineages in prehistoric sheep—lineages otherwise found only in ancient humans—indicating that sheep served as important intermediate hosts in human plague transmission for at least 4,000 years[10].This deep evolutionary context underscores the public health significance of monitoring plague in modern pastoral systems.However,despite the rapid expansion of Tibetan sheep populations in Gansu Province,the risk of plague infection and transmission in this species has not yet been systematically evaluated,leaving a significant research gap.This study addresses three specific questions:Do Tibetan sheep in Subei County exhibit serological evidence of *Y. pestis* exposure?Is the spatial

distribution of seropositive herds associated with active marmot plague detection sites? What ectoparasite species are present on Tibetan sheep in this region, and do they include taxa documented on Himalayan marmots?Based on the empirical findings, targeted risk mitigation strategies will be proposed to provide a scientific evidence base for decision-making,thereby facilitating the prevention and control of plague outbreaks and safeguarding public health and social stability.

Research Methods

Ethics Statement

This study was conducted in strict accordance with the guidelines for animal experimentation and was approved by the Ethics Committee of Gansu Provincial Center for Disease Control and Prevention(Gansu CDC Ethics [2025] No. 009).All procedures involving animals were performed following the Regulations on the Administration of Laboratory Animals of the People's Republic of China and the Guidelines for the Care and Use of Laboratory Animals.Prior informed consent was obtained from all herders/owners of the Tibetan sheep included in this study.The study protocol was designed to minimize animal suffering,and all sampling procedures were performed by trained veterinary professionals using standard aseptic techniques.No animals were euthanized for the purpose of this study.

Sample Collection

Sampling for this study was conducted during March-May 2024-2025(spring sampling, coinciding with marmot emergence and plague spread)and September–November 2024–2025(autumn sampling, coinciding with marmot hibernation and plague subsidence),The sampling area comprised Shibaocheng and Yanchiwan townships in Subei Mongolian Autonomous County,where field reconnaissance confirmed Tibetan sheep herds within the Himalayan marmot plague-endemic area.All nine villages with known Tibetan sheep populations in these two townships were included.Within each village,herders raising Tibetan sheep were approached based on accessibility and willingness to participate;not all herders in each village were sampled.From each participating household,all Tibetan sheep present on the sampling day were eligible for inclusion.Venous blood, nasal swabs, and external parasites were collected from Tibetan sheep within the study area.

Geographic coordinates of each sampling site were recorded at the center of the pasture using mobile positioning software (accuracy ± 5 m) and imported into ArcGIS 10.8 (ESRI, USA) for spatial analysis.

Sample Preparation

After collection,centrifuge venous blood at 2000 rpm for 10 minutes to separate the serum. Pipette the supernatant into sample storage tubes.After inactivation in a 56°C water bath for 30 minutes, the serum is ready for subsequent testing.Nasal swabs are collected and stored in bacterial preservation solution for testing.External parasites were collected by trained veterinary personnel via systematic visual inspection and manual combing using fine-toothed combs over wool-rich anatomical regions (neck, axilla, groin, and perineum) of each animal. Parasites were then removed with forceps, placed into collection tubes,and transported to the laboratory.After taxonomic

identification under a microscope using standard morphological keys, specimens were ground in PBS buffer for subsequent testing..

Sample Testing

Using the serum processed as described above,F1 antibody testing was performed in accordance with the operating instructions for the *Yersinia pestis* F1 Antibody Detection Kit and Appendix E of the Indirect Hemagglutination Assay (IHA) for Plague (WS279-2008) in the"Diagnostic Criteria for Plague"[11].Both nasal swabs and ground samples of external parasites were subjected to molecular biological testing and bacterial culture.For molecular biological testing,nucleic acids were first extracted using a tissue genomic DNA extraction kit,followed by PCR amplification(Primers are shown in Table 1) and electrophoresis.The amplification system for each sample consisted of 15 μl of 2× PCR Mix,0.5 μl of fraF, 0.5 μl of fraR, 0.5 μl of plaF, 0.5 μl of plaR, 1 μl of the sample to be tested,and 12 μl of nucleic acid-free water;the amplification conditions were 5 min at 95°C for pre-denaturation, followed by 30 cycles of 1 min at 95°C,1 min at 55°C, and 1 min at 72°C,with a final extension of 5 min at 72°C. Bacterial cultures were performed using Hersh medium at 28°C for at least 24 hours,after which colony growth and morphological characteristics were observed. All detection reagents are listed in Table 2..

Statistical Analysis

Data entry and summarization were performed using Excel;statistical analysis was conducted using SPSS 27.0 For data from multiple independent samples,Fisher's exact test and chi-square tests were performed. All statistical tests were two-tailed;$P < 0.05$ was considered statistically significant.Ectoparasite community diversity was quantified using the Shannon diversity index (H') and Simpson diversity index, calculated based on the number of individuals per taxon.Spatial analysis of relevant data was performed using ArcGIS 10.8.

Table 1 Target Gene Design

Gene Name	Primer Sequence	Product Length (bp)
Fra	F：GGAACCACTAGCACATCTGTT	249
	R：ACCTGCTGCAAGTTTACCGCC	
Pla	F：ACTACGACTGGATGAATGAAAATG	456
	R：GTGACATAATATCCAGCGTTAATT	

Table 2 Details of Sample Detection Reagents

Test Name	Equipment/Reagent		
	Name	Manufacturer	Batch Number
Plague Indirect Hemagglutination Assay (IHA)	Plague Antibody Detection Kit	Lanzhou Institute of Biological Products Co., Ltd.	20250301
Nucleic Acid Extraction	Tissue Genomic DNA Extraction Kit (CD170-01)	Lanzhou Meibo Biomedical Technology Co., Ltd.	CD17001250521
Polymerase Chain Reaction (PCR)	DL-1000 Marker	Takara Biotechnology Co., Ltd.	A1001B
	2×PCR MIX	Takara Biotechnology Co., Ltd.	A061983A
	GelRed Nucleic Acid Stain	Takara Biotechnology Co., Ltd.	20250314
	5×TBE Buffer	Beijing Solarbio Science & Technology Co., Ltd.	240001001
Bacterial Culture	Herrold's Medium	Beijing Luqiao Technology Co., Ltd.	250116

Results

Sample Collection

A total of 3,033 Tibetan sheep were sampled for this study,with 1,329 sampled in the spring and 1,704 in the autumn.The collected samples originated from Nan ning Guo le Village (926 sheep),Kui teng Guo le(185 sheep),and Diao er li ji(202 sheep) in Yan chi wan Township,Subei County; Jin gou(92 sheep) and Shi ban dun(32 sheep) in Shi bao cheng; Ying zui shan(16), Ha shi ha er(4), Yu er hong Pasture (1,102), and Yu er hong Village (474)—totaling 35 sheep herds from 9 villages (Figure 1,Table 3). Among these,4 herding sites were located at altitudes between 2,500 and 3,000 meters, while the remaining sites were situated between 3,000 and 4,000 meters.A total of 3,033 serum samples,1,090 nasal swab samples,and 36 Tibetan sheep (22 groups) were collected for examination of external parasites (Table 4).

Serological Detection

Results of the indirect hemagglutination test for plague showed that 10 of the 3,033 Tibetan sheep serum samples tested positive for plague F1 antibody,with a positivity rate of 3.3‰ (10/3,033).Given the observed seroprevalence of 3.3‰, the sample size of 3,033 provides >99% power to detect a prevalence of ≥1‰ at α=0.05 (two-tailed). However,the rarity of positive events (n=10) precluded multivariable risk factor analysis for secondary objectives.Among these, the highest seroprevalence of plague antibodies was found in Tibetan sheep from Jin gou,at 10.8‰ (1/92);followed by Yu er hong Pasture,with a seroprevalence of 5.4‰ (6/1102);and Nan ning guo le, with a

seroprevalence of 3.2‰ (3/926);no positive serum samples were detected in the remaining villages.Regarding the plague F1 antibody titers in the positive samples, titers ranged from 1:16 to 1:128, including 1 sample at 1:128, 1 at 1:64, 1 at 1:32, and 7 at 1:16.Overall, antibody titers were relatively low and distributed sporadically (Table 5). The 10 positive samples were collected from six different sheep flocks. Among them,five positive samples were detected in the flock of 387 Tibetan sheep belonging to Herder D,which had the highest positive rate of 13.0‰ (5/387);the positive rates for the flocks of the remaining five herders ranged from 2.5‰ to 9.5‰.

Table 3 Number of Tibetan Sheep Samples Collected in Suobei Mongol Autonomous County, Gansu Province

Township	Village	Spring Sampling		Autumn Sampling		Total	
		Number of Households	Households Sampled	Number of Households	Households Sampled	Number of Households	Households Sampled
Yan chi wan	Nan ning guo le	6	290	6	636	12	926
	Kui teng guo le	—	—	3	185	3	185
Shi bao cheng	Diao er li ji	3	202	—	—	3	202
	Jin gou	—	—	1	92	1	92
	Shi ban dun	—	—	2	32	2	32
	Ying zui mountain	—	—	1	16	1	16
	Ha shi ha er	2	4	—	—	2	4
	Yu er hong Ranch	3	819	1	283	4	1102
	Yu er hong	1	14	6	460	7	474
Total		15	1329	20	1704	35	3033

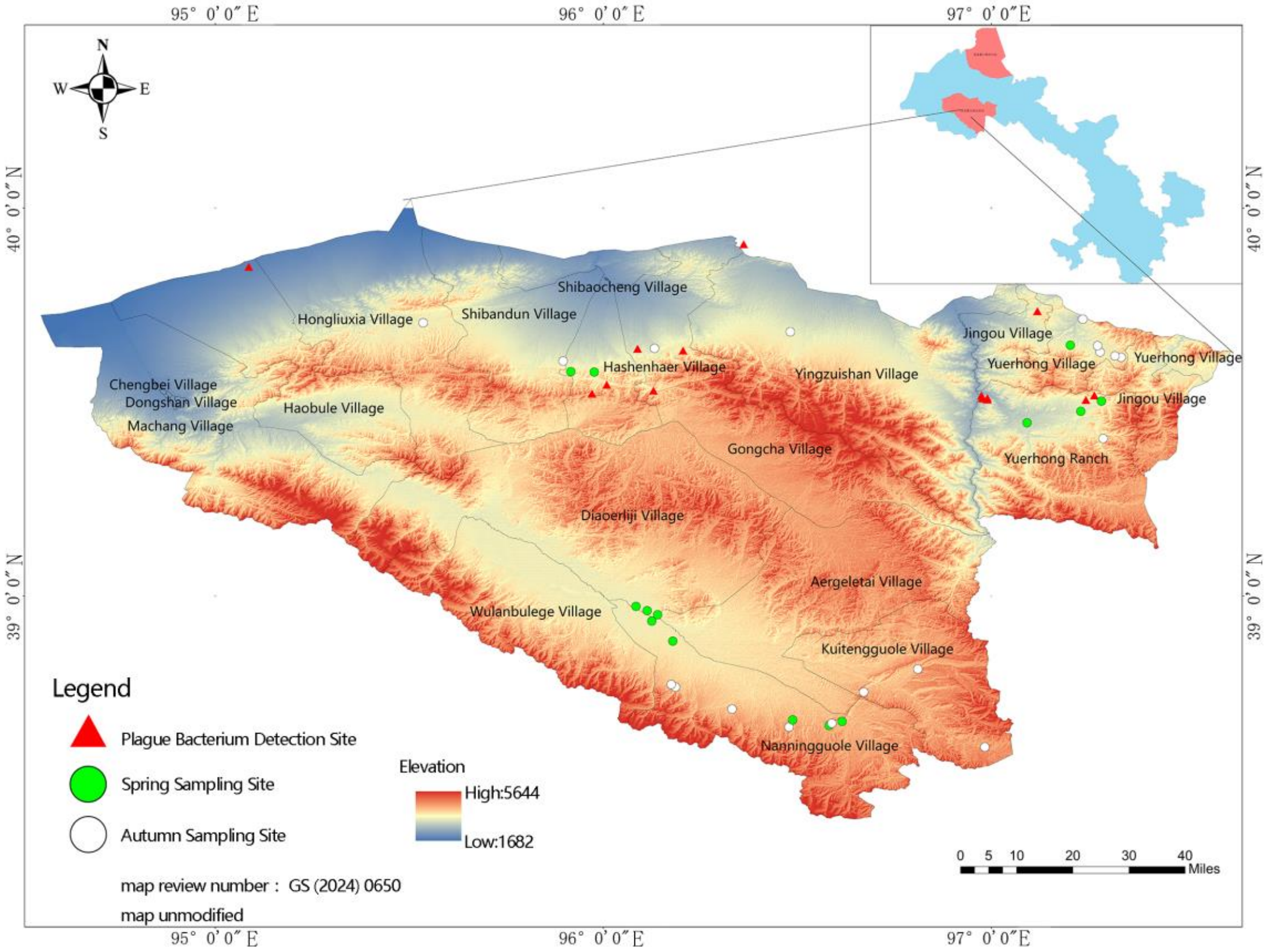

Figure 1 Distribution of Tibetan Sheep Sampling Sites and Plague Bacterium Detection Sites.Red triangles indicate plague bacterium detection sites, green circles indicate spring sampling sites, and open circles indicate autumn sampling sites. The administrative division basemap was obtained from Tianditu (https://cloudcenter.tianditu.gov.cn/administrativeDivision), map review number GS (2024) 0650. The map is unmodified.

Table 4 Distribution of Specimen Types by Village and Herd

Township	Village	Number of Households	Number of nasal swabs	Number of serum samples	Number of Ectoparasite samples
Yan chi wan	Nan ning guo le	12	245	926	34
	Kui teng guo le	3	—	185	2
	Diao er li ji	3	202	202	—
Shi bao cheng	Jin gou	1	—	92	—
	Shi ban dun	2	—	32	—
	Ying zui mountain	1	—	16	—
	Ha shi ha er	2	—	4	—

	Yu er hong Ranch	4	627	1102	—
	Yu er hong	7	16	474	—
Total		35	1090	3033	36

Table 5　Results of F1 Antibody Detection for Plague in Tibetan Sheep Sera

Region		Tested Samples	Positive Samples	Positive Rate	Positive Sample Herder ID	Positive Sample ID	Sample Titer	Positive Sample Season
Township	Village							
Yan chi wan	Nan ning guo le	926	3	3.2‰	A	Y1-74（W）	1：16	Autumn
					B	Y893	1：16	Spring
					C	Y1278	1：16	Autumn
Shi bao cheng	Yu er hong Ranch	1102	6	5.4‰	D	H503	1：32	Spring
					D	H635	1：16	Spring
					D	H670	1：16	Spring
					D	H785	1：16	Spring
					D	H807	1：128	Spring
					E	H918	1：64	Spring
Shi bao cheng	Jin gou	92	1	10.8‰	F	H2540	1：16	Autumn
Yan chi wan	Kui teng guo le	185	0	0‰	—	—	—	—
	Diao er li ji	202	0	0‰	—	—	—	—
Shi bao cheng	Shi ban dun	32	0	0‰	—	—	—	—
	Ying zui mountain	16	0	0‰	—	—	—	—
	Ha shi ha er	4	0	0‰	—	—	—	—
	Yu er hong	474	0	0‰	—	—	—	—
Total		3033	10	3.3‰				

Spatial Distribution

Combined with the results of *Yersinia pestis* detection from the same period in this epidemic area,spatial analysis showed that the median distance from *Yersinia pestis* detection sites to sites with positive *Yersinia pestis* F1 antibody results in Tibetan sheep serum (46,456m) was shorter than the median distance to sites with negative results (61,689m);however,the difference between the two groups was not statistically significant ($U=85$, $Z=-0.09$, $P=0.93$),suggesting that, given the current sample size, the spatial association analysis between seropositive Tibetan sheep and *Yersinia pestis* detection sites has not yet reached statistical significance.

Ectoparasite Detection

Combing examinations of Tibetan sheep for external parasites yielded a detection rate of 0.70% (21/3033).The 21 affected sheep were from five different flocks, with detection rates as follows:Flock B 0.70% (1/150),Flock C 2.30% (9/400),Flock G: 64.0% (7/11),Flock H: 2.24% (2/89),and Flock I: 4.0% (2/50).The differences in the detection rates of ectoparasites among the different herds were statistically significant (Fisher's Exact Test, $P < 0.001$).

A total of 36 ectoparasites were collected,with an overall parasite index of 0.012 (36/3,033).The breakdown by flock was:Flock B:2 parasites, index 0.013 (2/150); Flock C:17 parasites, index 0.043 (17/400);Flock G:13 parasites, index 1.182 (13/11); Flock H:2 parasites, index 0.022 (2/89);and Flock I:2 parasites, index 0.040 (2/50).

Seasonally,spring sampling (April–May) of 1,329 sheep identified 12 infested animals carrying 19 parasites,with a detection rate of 0.90% (12/1,329) and a parasite index of 0.014.Autumn sampling (October–November) of 1,704 sheep identified 9 infested animals carrying 17 parasites,with a detection rate of 0.53% (9/1,704) and a parasite index of 0.010.No statistically significant difference in ectoparasite detection rates was observed between seasons ($\chi^2 = 1.53$, $P > 0.050$).

Ectoparasites were identified using standard taxonomic keys.The community composition comprising 6 hard ticks (Ixodidae), accounting for 16.70% (6/36), and 30 mites (Acari), accounting for 83.30% (30/36) (Table 6).Ectoparasite community structure was characterized by low diversity.The Shannon diversity index (H') was 0.45 and the Simpson diversity index was 0.28,reflecting marked dominance of mites (83.3%) over ticks (16.7%).

Table 6 Taxonomic Identification of Ectoparasites on Tibetan Sheep

NO.	Township	Village	Herder ID	Tibetan Sheep ID	Vector Classification Quantity		Sample Collection Season	Total
					Hard Tick (Ixodidae)	Mite (Acari)		
1	Yan chi wan	Nan ning guo le	G	Y103		2	Spring	2
2			G	Y135		1	Spring	1
3			G	Y152		1	Spring	1
4			G	Y181		1	Spring	1
5			G	Y190		6	Spring	6
6			G	Y191		1	Spring	1
7			G	Y194	1		Spring	1
8			H	Y720	1		Spring	1
9			H	Y830	1		Spring	1
10			B	Y977		2	Spring	2
11		Diao er li ji	I	Y596	1		Spring	1
12			I	Y649	1		Spring	1
13		Nan ning guo le	C	Y1002		2	Autumn	2
14			C	Y1005		1	Autumn	1
15			C	Y1009		1	Autumn	1
16			C	Y1088		2	Autumn	2
17			C	Y1216		1	Autumn	1
18			C	Y1254		3	Autumn	3
19			C	Y1318	1	2	Autumn	3
20			C	Y1338		3	Autumn	3
21			C	Y1374		1	Autumn	1
Total					6	30		36

Note: Specimens were identified to family (Ixodidae) or subclass (Acari) level by morphological stereomicroscopy using standard taxonomic keys.

Molecular and Culture Results

In this study, 1,090 nasal swab samples and 36 parasites (22 groups) were subjected to both molecular biological testing and bacterial culture. The results showed that no corresponding positive bands were observed in the electrophoresis following PCR amplification, indicating that all samples were negative; furthermore, no colonies

characteristic of *Yersinia pestis* were observed in the bacterial cultures, indicating that all samples were negative.

Discussion

As one of China's three major coarse-wooled sheep breeds, the Tibetan sheep is an ancient indigenous breed widely distributed across high-altitude regions of the Qinghai-Tibet Plateau, including Tibet, Qinghai, Gansu, Yunnan, and Sichuan. Leveraging its geographical advantages as a highland interior and vast pasture resources, Qinghai Province has gradually developed into a core production area for Tibetan sheep. However, in recent years, Gansu Province has introduced Tibetan sheep to improve its sheep breed structure and increase production, and the scale of its herd is gradually expanding.

The grazing sites in this study spanned 2,500-4,000 m, directly overlapping the core habitat of the Himalayan marmot (2,900-5,500 m) [12], creating ecological conditions for cross-species *Yersinia pestis* transmission. Although a small number of marmot populations may descend along river valleys to riverbank grasslands at around 2,500 meters or to field ridges at around 2,800 meters, their core distribution area significantly overlaps spatially with the grazing areas of Tibetan sheep. This high degree of ecological niche overlap greatly increases the opportunities for contact between Tibetan sheep grazing areas and Himalayan marmots within the same geographical unit, creating natural conditions for the cross-species transmission of *Yersinia pestis*.

The 3.3‰ seroprevalence observed in this study aligns with the 0.9‰–10.7‰ (average 4.7‰) reported from multi-site surveillance in Qinghai Province[6], suggesting that plague exposure in Tibetan sheep is a stable component of the regional enzootic cycle rather than an isolated incident.Furthermore, by combining the patterns of antibody fluctuation in Tibetan sheep [6], the antibody half-life, and the 2-fold decay rule, it can be roughly estimated that the infection occurred between April 2024 and October 2025, which closely aligns with the marmot activity season (April–October annually). As an introduced species, Tibetan sheep have a relatively short history of grazing in the Jiuquan region of Gansu, yet the positivity rate has already reached 3.3‰. This indicates that once introduced into the epidemic focus, Tibetan sheep can be rapidly exposed to *Yersinia pestis* through direct contact, shared pastures, and vector bites. Although no human cases have been detected to date—likely due to factors such as the short duration of Tibetan sheep rearing, limited human contact, and the distance between marmot habitats and residential areas—this epidemic focus possesses a complete "marmot-Tibetan sheep-human" transmission chain.Human cases in this region have historically been linked to marmots and sheepdogs rather than sheep, likely due to closer human-dog contact and the potential for dogs to act as mechanical carriers or bridge hosts.Tibetan sheep may pose risk primarily through contact with infected carcasses or products (wool, meat) rather than direct zoonotic transmission[13].Without proactive surveillance and an immunological barrier among livestock, activation of this chain would significantly increase the risk of sporadic or even clustered human outbreaks.

Vector organisms play a key role in transmitting pathogens within the plague transmission chain. The ectoparasite index was 0.012. No fleas (Siphonaptera) were recovered in this survey. The ectoparasites identified were hard ticks (6/36, 16.7%) and mites (30/36, 83.3%) (Table 6).The low yield likely reflects the cross-sectional design with spring and autumn sampling, which may have missed peak vector activity; additionally, some herders had recently applied acaricide dipping prior to our arrival..The ectoparasite index is influenced by various factors such as season, climate, and host density [14-15]; a single-season cross-sectional survey may not fully reflect vector dynamics. Future research should conduct continuous monitoring across multiple seasons and regions to obtain more reliable vector density data. Regarding the types of parasites detected, although the detection of the hard ticks (Ixodidae) and mites (Acari) does not indicate they are dominant vectors on the body surface of the Himalayan marmot, they are among its primary parasitic vectors. Furthermore, surveys in natural foci have confirmed that these vectors can carry the pathogen responsible for plague [16]. The detection of these two types of parasites ecologically confirms that vector-host switching does indeed occur between marmots and Tibetan sheep. When marmots die from plague, these parasites detach and re-parasitize the bodies of Tibetan sheep. Although the duration of parasitism may be brief, during plague outbreaks, this transient host switching is sufficient to facilitate interspecies transmission of the pathogen.

Molecular biology and bacterial culture tests of nasal swabs and parasite samples were all negative. Concurrently conducted positive and negative controls yielded corresponding results, indicating that the test results are reliable. Possible reasons for the negative sample results include: low pathogen load in the samples, below the limit of detection, resulting in negative results; studies have shown that different animal species exhibit significant differences in their response to *Yersinia pestis* infection, with some animals potentially presenting as subclinical infections or transient carriers [17]. If the sampling timing did not cover the peak period of bacterial shedding, it would be difficult to detect the pathogen. However, in conjunction with the results of the serological survey conducted concurrently with this study (3.3‰), it can be inferred that Tibetan sheep do indeed have a history of exposure to *Yersinia pestis*; however, pathogen evidence may not have been detected due to rapid clearance of the pathogen, inappropriate sampling timing, or limitations in the sensitivity of the detection method. This coexistence of serological positivity and negative pathogen detection is not uncommon in the surveillance of naturally occurring zoonotic diseases, suggesting that Tibetan sheep may primarily exhibit a “rapid clearance after infection” host pattern rather than a state of long-term carriage. Tibetan sheep may serve as “terminal hosts” or “sentinel animals” rather than “reservoir hosts.” Although they rapidly clear the pathogen after infection, they retain antibodies, which in turn confirms the activity of the epidemic focus; however, they themselves may not contribute to maintaining the focus. This distinction is crucial for the formulation of control strategies.

Limitations

This study has several limitations. First, demographic data (age, sex, and reproductive status) were not systematically recorded, preventing host-level risk factor analysis and potentially introducing bias if susceptibility to plague infection or ectoparasite infestation varies by these factors. Second, the cross-sectional design precludes inference of temporal dynamics in vector exposure and seroconversion; longitudinal monitoring across multiple seasons is needed. Third, the low sensitivity of bacterial culture and the potential for transient pathogen carriage below the PCR detection limit may have yielded false negatives for active infection. Fourth, the spatial analysis did not reach statistical significance ($U=85$, $Z=-0.09$, $P=0.93$), likely due to limited sample size and the broad geographic scale of the foci; finer-scale GPS tracking of individual animals would strengthen future spatial epidemiological models. Finally, ectoparasite identification was limited by the absence of molecular barcoding; future studies should integrate DNA sequencing for definitive taxonomic assignment.

Conclusion

In summary, there is a risk of plague infection and transmission among Tibetan sheep and their vectors in the natural plague foci of the Himalayan marmot in Gansu Province. This study proposes the following targeted control measures: It is recommended that Tibetan sheep be included in the plague host surveillance system, with a focus on sampling and monitoring during plague outbreaks in marmots in endemic areas. Random sampling should be conducted on Tibetan sheep serum in pastoral areas, as well as at cattle and sheep trading markets and related biological materials. Serological and molecular biological methods should be used to detect plague F1 antibodies in the samples; Strengthen health education and behavioral interventions for high-risk groups (herders, sheep traders, and slaughterhouse workers) to enhance their awareness of and capacity for protection, thereby safeguarding their health. Concurrently, in-depth basic research on Tibetan sheep in plague-endemic areas should be conducted, exploring aspects such as infection mechanisms to provide a theoretical basis for optimizing plague prevention and control strategies.

References

[1]Wang Tiefeng, Guan Xin, Wang Zhao. Research status of detection techniques for Yersinia pestis [J]. Chinese Journal of Endemic Disease Control, 2025, 40(02): 115-117.

[2]Wang X, Jing HQ, Kan B. Long and arduous road: prevalence and prevention of plague in modern society. Chinese Journal of Tropical Medicine, 2024, 24(1):22-27+48.

[3]Qin CY, Xu L, Zhang RZ, et al. Typing of natural plague foci in China V: biological characteristics of plague hosts. Chinese Journal of Epidemiology, 2012, 33(7):692-697.

[4]Xiong HM, Jin J, Xin YQ, et al. Phylogenetic relationship and epidemiological characteristics of Yersinia pestis isolated from Tibetan sheep on the Qinghai Plateau. Chinese Journal of Disease Control & Prevention, 2022, 26(12):1473-1478. DOI:10.16462/j.cnki.zhjbkz.2022.12.019.

[5]Tang XY. Epidemiological analysis of plague in Tibetan sheep in Qinghai province from 1975 to 2015[J]. Chinese Qinghai Journal of Animal and Veterinary Sciences, 2016, 46(5): 19-21.

[6]Yu DZ. Animal Epidemiology of Plague. Beijing: Science Press, 2009:263-271.

[7]Tian FZ. Natural plague foci of Himalayan marmot [J]. Chinese Journal of Zoonoses, 2000, 16(4): 95-96.

[8]Cong XB, Ju C. Human Plague in China. Beijing: People’s Medical Publishing House, 2018:92-104.

[9]Cai LQ, Kan GQ, Ma L, et al. Current status of animal husbandry development and paths for cultivating superior and characteristic industries in Subei County. Gansu Animal Husbandry and Veterinary Medicine, 2024, 54(5):128-132. DOI:10.15979/j.cnki.cn62-1064/s.2024.05.002.

[10]Light-Maka I, Hermes T R, Bianco R A, et al. Bronze Age Yersinia pestis genome from sheep sheds light on hosts and evolution of a prehistoric plague lineage[J]. Cell, 2025, 188(20): 5748-5762. e18.

[11]Ministry of Health of the People’s Republic of China. Diagnostic criteria for plague (WS279-2008). Beijing: Standards Press of China, 2008:10-11.

[12]Bao ZQ, Li C, Guo C, et al. Convergent evolution of Himalayan marmot with some high-altitude animals through ND3 protein. Animals (Basel), 2021, 11(2):251.

[13]Dai RX, Yang XY, Yang YH, et al. Study on the pathogenic ecology of plague in Tibetan sheep on the Qinghai Plateau and its epidemiological significance. Chinese Journal of Endemiology, 2014, 33(5): 492-494.

[14] Li ZL, Wang CG, Ma LM. Relationship between density of Spermophilus dauricus, meteorological factors and flea index. Chinese Journal of Vector Biology and Control, 1993, (4):282-283.

[15]Wang DD, Xu DQ, Ge PF, Xi JX, et al. Relationship between the number of parasitic fleas on *Spermophilus alashanicus*, host abundance and meteorological factors. *Chinese Journal of Endemiology*, 2018, 37(12):965-968.

[16]Li HL, Wei YW, Li C, et al. Cluster analysis of medical gamasid mites in Qinghai Province. *Chinese Journal of Zoonoses*, 2014, 30(1):67-73.

[17]Chen M, Shi LY. Research progress on susceptibility, resistance and mechanisms of plague-infected animals to *Yersinia pestis*. *Chinese Journal of Endemiology*, 2023, 42(7):593-597.

Figure

Click here to access/download;Figure;Figure 1.tif

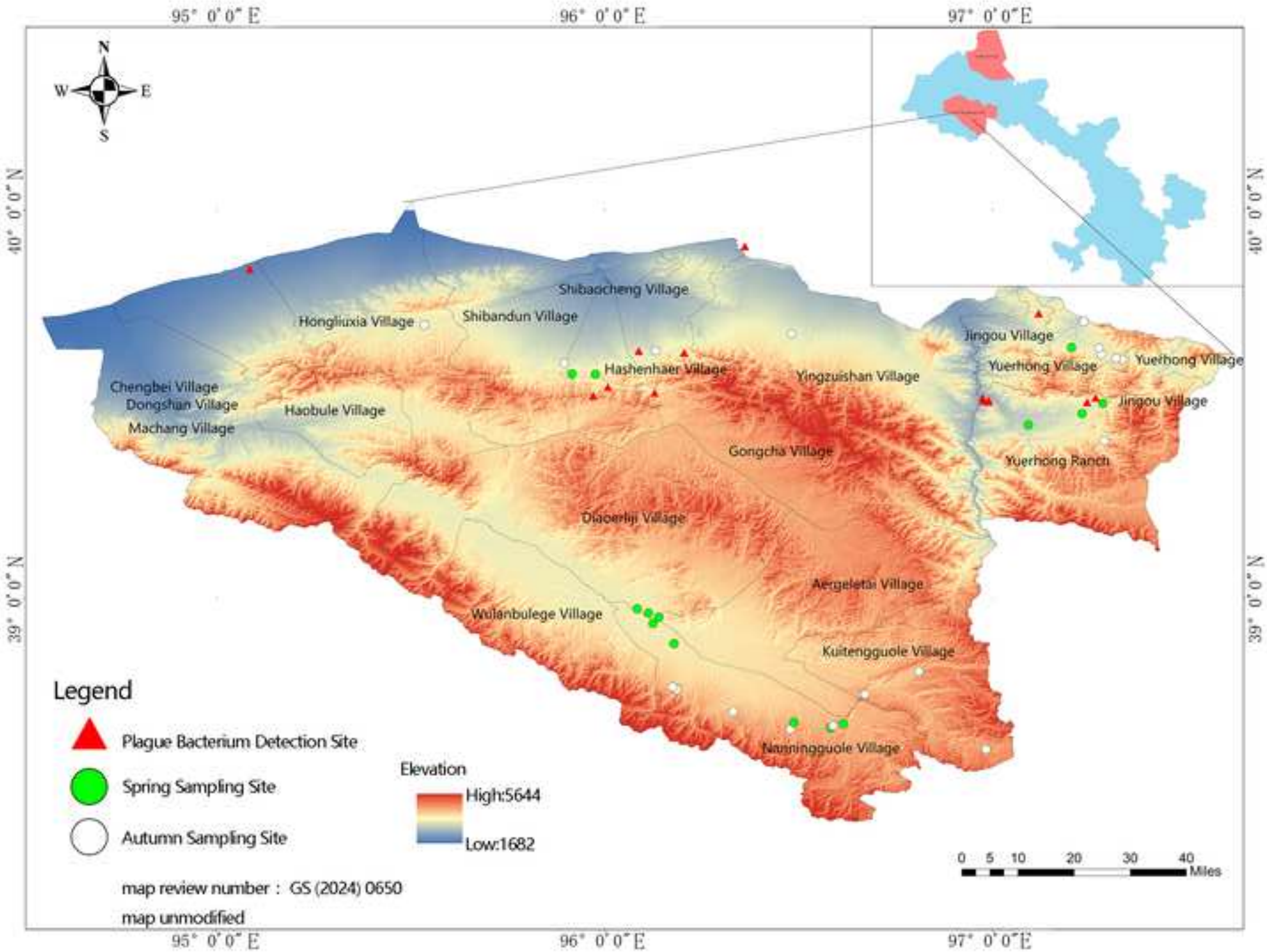

Dear Editor and Reviewers,

Author Contributions Statement:We wish to clarify that this manuscript has six co-first authors: Wenjing An, Aiwei He, Guotian Ma, Hua Chun, Geli Aoer, and Dingsheng Wang.These authors contributed equally to this work.The corresponding author is Daqin Xu(Email: 317645180@qq.com),who conceived and supervised the study.All changes in the revised manuscript are highlighted with tracked changes.

Thank you for your constructive and detailed comments on our manuscript. We have carefully revised the manuscript to address all points. Below, we provide a point-by-point response to each comment. All changes in the revised manuscript are highlighted with tracked changes.

Editor’s Requirements

1. CRediT author contributions

Response:We have completed the CRediT taxonomy for all 15 authors in the submission system. A summary table is provided below for reference.

2. Author Summary

Response:We have added an Author Summary (186 words) between the Abstract and the Introduction, written for a broad audience including non-scientists.

3. Ethics approval number

Response:We have inserted the ethics approval number (Approval No. Gan CDC Ethics (2025) 009) into the Ethics Statement in the Methods section.

4.Financial Disclosure

Response:We have rewritten the disclosure in full sentences, specifying the initials of authors receiving each grant, grant numbers, full funder names, and confirming that the funders had no role in the study.

Reviewer #1

Comment 1:Provide more information about the trends in introduction and distribution of Tibetan sheep within the known plague endemic areas.

Response:We have added official county statistics showing that the sheep inventory in Subei County increased from 337,860 head in 2020 to 414,304 head in 2022 , and stated that according to field surveys, Tibetan sheep—a confirmed secondary host of plague—have also increased in number through introduction.

Comment 2:The absence of information regarding the geographical distribution of the samples and their proximity to manifested plague makes the analysis less representative.

Response: We have included a map (Figure 1) showing the geographic distribution of sampled herds relative to known marmot plague detection sites.

Comment 3:The total number of ectoparasites (30 samples obtained from >3,000 sheep) raises the question about collection technique. The procedure for collecting ectoparasites is absent. It could be challenging to collect fleas, sheep flies, and other ectoparasites from sheep. This question should be addressed.

Response: We have added a detailed protocol in the Methods : “External parasites were collected by trained veterinary personnel via systematic visual inspection and manual combing using

fine-toothed combs over wool-rich anatomical regions (neck, axilla, groin, and perineum) of each animal. Parasites were then removed with forceps, placed into collection tubes, and transported to the laboratory.After taxonomic identification under a microscope using standard morphological keys, specimens were ground in PBS buffer for subsequent testing."

Regarding the low ectoparasite yield (36 specimens from 3,033 sheep, detection rate 0.7%), we acknowledge that this may reflect both genuine low infestation and methodological limitations. We note the following:

(1) Collection method: Manual combing and visual inspection, while standardized, may underestimate mobile ectoparasites such as fleas, which can rapidly evade detection in dense wool. This is a recognized limitation of non-immersion combing techniques;

(2) Timing and herd management: This was a cross-sectional survey conducted in spring and autumn; the low yield likely reflects both seasonal low vector activity and recent acaricide dipping by some herders prior to sampling.

Comment 4: It is essential to discuss and present the target gene used for molecular screening.*
Response: We have added Table 1 (Target Gene Design) in the Methods, detailing the two target genes: the fra gene (encoding F1 capsular antigen) and the pla gene (encoding plasminogen activator). The table includes Gene Name,Primer Sequence and Product Length.

Reviewer #2
Comment 1: The objectives lack a clear testable hypothesis. The introduction would benefit from a formal hypothesis.
Response:We appreciate this comment and have clarified the nature of our study in the revised Background. This study is exploratory and descriptive rather than hypothesis-driven. Its primary aim was to determine whether Tibetan sheep in Gansu Province's Himalayan marmot plague foci show evidence of Y. pestis exposure, following reports of seropositive Tibetan sheep in Qinghai Province and the recent expansion of sheep populations in Gansu. Accordingly, we have reframed the study objectives as three research questions: (1) Do Tibetan sheep in the Subei County plague foci exhibit detectable Y. pestis F1 antibodies? (2) Is the spatial distribution of seropositive animals associated with active marmot plague detection sites? (3) What ectoparasite species are present on Tibetan sheep in this region, and do they include taxa capable of interspecies host-switching from marmots? These questions were addressed through a multi-modal cross-sectional survey integrating serology, molecular biology, and spatial epidemiology. We believe this framing more accurately reflects the exploratory nature of the work.
Comment 2: The study design lacks a longitudinal component and sufficient methodological detail on herd selection.
Response: We have clarified the design: "This study represents a baseline cross-sectional survey conducted during spring and autumn to align with marmot activity peaks. Longitudinal vector dynamics will require future continuous monitoring." For herd selection, we have added: "All villages with known Tibetan sheep populations in Shibaocheng and Yanchiwan townships were included (nine villages total). Within each village, herders raising Tibetan sheep were approached based on accessibility and willingness to participate; not all herders in each village were sampled. From each participating household, all Tibetan sheep present on the sampling day were eligible

for inclusion." This approach was designed to maximize coverage of Tibetan sheep in the endemic area while acknowledging practical constraints of field access and herder availability.

Comment 3: The description lacks essential demographic data (age, sex, reproductive status).

Response: We acknowledge this limitation. Demographic data (age, sex, and reproductive status) were not systematically recorded during field sampling, as the primary objective was to assess population-level plague exposure rather than host-level risk factors. This is now explicitly stated in the Limitations paragraph: "Demographic data (age, sex, and reproductive status) were not systematically recorded, which prevents host-level risk factor analysis and may introduce bias if susceptibility to plague infection or ectoparasite infestation varies by these factors." We agree that future studies should incorporate demographic recording to enable stratified analysis of seroprevalence and vector burden by age, sex, and reproductive status.

Comment 4: The sample size is impressive, but the study lacks a formal power analysis.

Response: We appreciate the recognition of our sampling effort. We have added a post-hoc power statement in the Results: "Given the observed seroprevalence of 3.3‰, the sample size of 3,033 provides >99% power to detect a prevalence of ≥1‰ at α=0.05 (two-tailed)." We acknowledge that this study was designed as an exploratory prevalence survey rather than a hypothesis-driven analytical study. The secondary objectives—such as identifying environmental predictors of low-titer seroprevalence—were not powered a priori due to the rarity of positive events (n=10). Multivariable analysis of environmental covariates with only 10 positive cases would violate the rule of 10 events per variable and risk severe overfitting.

Comment 5: The statistical analysis is too shallow. Should use Logistic Regression/GLMs and diversity indices. Also, ArcGIS methodology is not supported by GPS data collection details.

Response:

Regression: We explain that multivariable logistic regression was not feasible because only 10 positive events were observed, violating the rule of 10 events per variable and risking severe overfitting. Instead, we performed univariate Fisher's exact tests and descriptive epidemiological summaries.

Diversity indices: We have calculated and added the Shannon diversity index (H′ = 0.45) and Simpson diversity index (0.28) for the ectoparasite community in the Results, reflecting the marked dominance of mites (83.3%) over ticks (16.7%). The calculation method has been added to the Methods.

GPS/ArcGIS:GPS data collection is described in the Methods (Sample Collection):"Geographic coordinates of each sampling site were recorded at the center of the pasture using mobile positioning software (accuracy ±5 m) and imported into ArcGIS 10.8 (ESRI, USA) for spatial analysis."

Comment 6: The conclusion regarding vector-borne transmission risk is weakened by a discrepancy: the flea index is mentioned, but Table 4 lists only ticks and mites.

Response: This is a critical correction. We have removed all mention of "flea index" throughout the manuscript and replaced it with "ectoparasite index" (0.012). We have revised Table 6(formerly Table 4) to accurately reflect the identified taxa:hard ticks (Ixodidae) and mites (Acari). We explicitly state: "No fleas (Siphonaptera) were recovered in this survey."

Comment 7: The limitations of the analysis are not clearly described.

Response: We have added a dedicated Limitations subsection in the Discussion, addressing five key constraints: (1) missing demographic data, which prevents host-level risk factor analysis; (2)

cross-sectional design, which precludes inference of temporal dynamics; (3) low sensitivity of culture/PCR for transient carriers, which may yield false negatives; (4) non-significant spatial analysis, likely due to limited seropositive samples and broad geographic scale; and (5) absence of molecular barcoding for ectoparasites, which limits definitive taxonomic assignment.

Comment 8: The Discussion restates large portions of raw data from the Results.

Response: We have removed redundant numerical restatements from the Discussion. The revised Discussion focuses on ecological interpretation, public health implications, and comparison with Qinghai Province data.

Comment 9: Yersinia pestis should be italicised throughout, including Tables and Figure captions.

Response: We have checked the entire manuscript and ensured that Yersinia pestis and Y. pestis appear in italics in all sections, tables, and figure captions.

Comment 10: Results should be restructured into subsections; a spatial map should be included; Table 4 needs genus/species.

Response:

- Results are now divided into five subsections: (1) Sample Collection. (2) Serological Detection.(3)Spatial Distribution. (4) Ectoparasite Detection.(5)Molecular and Culture Results.

-Figure 1 (ArcGIS map) has been prepared showing altitudinal overlap and proximity to marmot foci.

- Table 6 (formerly Table 4) has been revised. we have added a footnote: “Note: Specimens were identified to family (Ixodidae) or subclass (Acari) level by morphological stereomicroscopy using standard taxonomic keys.”

Reviewer #3

Comment 1: The Abstract should prominently state the effort: 3,033 sheep, 35 herds, 9 villages.

Response: The Abstract has been revised. The first sentence of the Results now reads: “A total of 3,033 Tibetan sheep from 35 herds across 9 villages were sampled...”

Comment 2:A map visualising the different herds/villages and pasture geography would be a great addition.

Response: We have prepared Figure 1, an ArcGIS map showing village locations, herd sampling sites (positive vs. negative), and the geography of pastures relative to Himalayan marmot habitat zones.

Comment 3: Nasal swabs are used for PCR. Is that established? Why not blood PCR?

Response: Nasal swabs were selected as the primary specimen for active infection screening based on the national diagnostic standard WS279-2008, which recommends respiratory/lesion materials for pathogen isolation in live animals." Blood PCR was not performed because serological positivity indicates past exposure rather than active bacteremia. In surveillance of naturally occurring zoonotic diseases, nasal/respiratory specimens are preferred for detecting active infection in live animals, while blood is typically used for serological evidence of exposure.

Comment 4: Only 1,090 samples were PCR tested. Why not 3,033? A table showing which specimen was tested from which herd may help.

Response: We have added Table 4 (Distribution of Specimen Types by Village and Herd). “Due to resource constraints and the prioritization of pathogen detection in high-risk villages, nasal swabs were collected from 1,090 sheep (35.9% of total), while serum was collected from all 3,033 sheep to maximize serological coverage.”

Comment 5: Is the 0.3% prevalence the true rate, or do infected animals die and become removed? What does infection mean for sheep health?

Response: We clarify that 0.33% (10/3,033) is the seroprevalence of F1 antibodies, not the infection rate—it reflects historical exposure to Y. pestis rather than active infection. All 10 seropositive animals were clinically healthy at sampling, and no plague-related deaths were reported by herders or observed during the study period. However, we acknowledge two limitations:(1) Potential underestimation due to mortality: Our cross-sectional design cannot exclude the possibility that acutely infected sheep died and were removed from the population before sampling. This is a recognized challenge in prevalence studies of lethal pathogens.(2) Health impact: Available evidence from Qinghai Province suggests Tibetan sheep typically develop subclinical or mild infections with rapid pathogen clearance, consistent with their role as secondary/sentinel hosts rather than reservoir hosts. The low antibody titers (1:16–1:128) and absence of clinical signs in our seropositive animals support this pattern. Severe disease or death appears rare, though carcass handling poses zoonotic risk to humans.

Comment 6: Why are the few human cases described presumably caused by rodents or sheepdogs but not sheep? What is the implication for human health?

Response: As an even-toed ungulate (Artiodactyla), the Tibetan sheep exhibits significant differences from primary reservoir hosts such as rodents in terms of susceptibility to *Yersinia pestis* and the pathological course following infection. Current research indicates that interspecies responses to plague infection are highly species-specific: while some animals may manifest as asymptomatic carriers or transient bacteremic states, the immune system of Tibetan sheep likely possesses robust innate clearance capacity against *Y. pestis*. Upon infection, Tibetan sheep can rapidly mount specific adaptive immune responses, facilitating pathogen elimination within a relatively short timeframe, thereby precluding the establishment of persistent, high-level bacteremia.This biological characteristic accounts for the epidemiological observation in Qinghai Province, where only 14 *Y. pestis* strains were isolated from Tibetan sheep between 1975 and 2015 (mean isolation rate: approximately 0.35 strains/year). Despite the low bacterial carriage and isolation rate, serological surveillance of Tibetan sheep retains substantial public health monitoring value—Tibetan sheep can serve as”plague sentinel animals”, whose dynamic seroprevalence patterns effectively predict enzootic trends, providing scientific evidence for early warning and prevention strategies.

Comment 7: The introduction relies on a very low number of references.

Response: We have expanded the Introduction with additional foundational literature on Y. pestis ecology, Tibetan sheep epidemiology, and vector biology. We have also added the suggested citation regarding ancient Y. pestis lineages in sheep (see below).

Comment 8:Cite PMID: 40795857 on ancient Y. pestis lineage in sheep.

Response: We thank the reviewer for this excellent suggestion. We have added the following to the Background: "The role of sheep in plague ecology extends beyond contemporary transmission dynamics. A recent study identified a Bronze Age Y. pestis genome in a 4,000-year-old domesticated sheep from the Eurasian Steppe, belonging to the Late Neolithic Bronze Age (LNBA) lineage previously known only from ancient humans [10]. This lineage lacked the genetic toolkit for efficient flea-borne transmission, suggesting sheep served as important intermediate hosts in prehistoric plague spread. This deep evolutionary context underscores the public health significance of monitoring plague in modern pastoral systems." This citation

strengthens the rationale for our study by connecting modern Tibetan sheep surveillance to millennia of sheep-plague co-evolution.

Click here to access/download
Revised Article with Changes Highlighted
Revised Manuscript with Track Changes.docx
