# Supplementary material for: A study on Tibetan sheep in the Gansu plague-endemic area and the risk of plague transmission by their vectors
Source: PLoS Negl Trop Dis. 2026 Aug 14;20(8):e0014650. doi: 10.1371/journal.pntd.0014650 (PMC13492742; doi:10.1371/journal.pntd.0014650)
Supplement: Supplementary file 3 [file pntd.0014650.s002.docx]

Dear Editor and Reviewers,

Author Contributions Statement:We wish to clarify that this manuscript has six co-first authors: Wenjing An, Aiwei He, Guotian Ma, Hua Chun, Geli Aoer, and Dingsheng Wang.These authors contributed equally to this work.The corresponding author is Daqin Xu(Email: [317645180@qq.com](mailto:317645180@qq.com)),who conceived and supervised the study.All changes in the revised manuscript are highlighted with tracked changes.

Thank you for your constructive and detailed comments on our manuscript. We have carefully revised the manuscript to address all points. Below, we provide a point-by-point response to each comment. All changes in the revised manuscript are highlighted with tracked changes.

**Editor’s Requirements**

1. CRediT author contributions

Response:We have completed the CRediT taxonomy for all 15 authors in the submission system. A summary table is provided below for reference.

2. Author Summary

Response:We have added an Author Summary (186 words) between the Abstract and the Introduction, written for a broad audience including non-scientists.

3. Ethics approval number

Response:We have inserted the ethics approval number (Approval No. Gan CDC Ethics (2025) 009) into the Ethics Statement in the Methods section.

4.Financial Disclosure

Response:We have rewritten the disclosure in full sentences, specifying the initials of authors receiving each grant, grant numbers, full funder names, and confirming that the funders had no role in the study.

**Reviewer #1**

**Comment 1**:Provide more information about the trends in introduction and distribution of Tibetan sheep within the known plague endemic areas.

**Response**:We have added official county statistics showing that the sheep inventory in Subei County increased from 337,860 head in 2020 to 414,304 head in 2022 , and stated that according to field surveys, Tibetan sheep—a confirmed secondary host of plague—have also increased in number through introduction.

**Comment 2**:The absence of information regarding the geographical distribution of the samples and their proximity to manifested plague makes the analysis less representative.

**Response:**We have included a map (Figure 1) showing the geographic distribution of sampled herds relative to known marmot plague detection sites.

**Comment 3**:The total number of ectoparasites (30 samples obtained from >3,000 sheep) raises the question about collection technique. The procedure for collecting ectoparasites is absent. It could be challenging to collect fleas, sheep flies, and other ectoparasites from sheep. This question should be addressed.

**Response:**We have added a detailed protocol in the Methods : “External parasites were collected by trained veterinary personnel via systematic visual inspection and manual combing using fine-toothed combs over wool-rich anatomical regions (neck, axilla, groin, and perineum) of each animal. Parasites were then removed with forceps, placed into collection tubes, and transported to the laboratory.After taxonomic identification under a microscope using standard morphological keys, specimens were ground in PBS buffer for subsequent testing.”

Regarding the low ectoparasite yield (36 specimens from 3,033 sheep, detection rate 0.7%), we acknowledge that this may reflect both genuine low infestation and methodological limitations. We note the following:

(1) Collection method: Manual combing and visual inspection, while standardized, may underestimate mobile ectoparasites such as fleas, which can rapidly evade detection in dense wool. This is a recognized limitation of non-immersion combing techniques;
(2) Timing and herd management: This was a cross-sectional survey conducted in spring and autumn; the low yield likely reflects both seasonal low vector activity and recent acaricide dipping by some herders prior to sampling.

**Comment 4:**It is essential to discuss and present the target gene used for molecular screening.*

**Response:** We have added Table 1 (Target Gene Design) in the Methods, detailing the two target genes: the fra gene (encoding F1 capsular antigen) and the pla gene (encoding plasminogen activator). The table includes Gene Name,Primer Sequence and Product Length.

**Reviewer #2**

**Comment 1:**The objectives lack a clear testable hypothesis. The introduction would benefit from a formal hypothesis.

**Response**:We appreciate this comment and have clarified the nature of our study in the revised Background. This study is exploratory and descriptive rather than hypothesis-driven. Its primary aim was to determine whether Tibetan sheep in Gansu Province's Himalayan marmot plague foci show evidence of Y. pestis exposure, following reports of seropositive Tibetan sheep in Qinghai Province and the recent expansion of sheep populations in Gansu. Accordingly, we have reframed the study objectives as three research questions: (1) Do Tibetan sheep in the Subei County plague foci exhibit detectable Y. pestis F1 antibodies? (2) Is the spatial distribution of seropositive animals associated with active marmot plague detection sites? (3) What ectoparasite species are present on Tibetan sheep in this region, and do they include taxa capable of interspecies host-switching from marmots? These questions were addressed through a multi-modal cross-sectional survey integrating serology, molecular biology, and spatial epidemiology. We believe this framing more accurately reflects the exploratory nature of the work.

**Comment 2:**The study design lacks a longitudinal component and sufficient methodological detail on herd selection.

**Response:**We have clarified the design: "This study represents a baseline cross-sectional survey conducted during spring and autumn to align with marmot activity peaks. Longitudinal vector dynamics will require future continuous monitoring." For herd selection, we have added: "All villages with known Tibetan sheep populations in Shibaocheng and Yanchiwan townships were included (nine villages total). Within each village, herders raising Tibetan sheep were approached based on accessibility and willingness to participate; not all herders in each village were sampled. From each participating household, all Tibetan sheep present on the sampling day were eligible for inclusion." This approach was designed to maximize coverage of Tibetan sheep in the endemic area while acknowledging practical constraints of field access and herder availability.

**Comment 3:**The description lacks essential demographic data (age, sex, reproductive status).

**Response:**We acknowledge this limitation. Demographic data (age, sex, and reproductive status) were not systematically recorded during field sampling, as the primary objective was to assess population-level plague exposure rather than host-level risk factors. This is now explicitly stated in the Limitations paragraph: "Demographic data (age, sex, and reproductive status) were not systematically recorded, which prevents host-level risk factor analysis and may introduce bias if susceptibility to plague infection or ectoparasite infestation varies by these factors." We agree that future studies should incorporate demographic recording to enable stratified analysis of seroprevalence and vector burden by age, sex, and reproductive status.

**Comment 4:**The sample size is impressive, but the study lacks a formal power analysis.

**Response:**We appreciate the recognition of our sampling effort. We have added a post-hoc power statement in the Results: "Given the observed seroprevalence of 3.3‰, the sample size of 3,033 provides >99% power to detect a prevalence of ≥1‰ at α=0.05 (two-tailed)." We acknowledge that this study was designed as an exploratory prevalence survey rather than a hypothesis-driven analytical study. The secondary objectives—such as identifying environmental predictors of low-titer seroprevalence—were not powered a priori due to the rarity of positive events (n=10). Multivariable analysis of environmental covariates with only 10 positive cases would violate the rule of 10 events per variable and risk severe overfitting.

**Comment 5:**The statistical analysis is too shallow. Should use Logistic Regression/GLMs and diversity indices. Also, ArcGIS methodology is not supported by GPS data collection details.

**Response:**

Regression: We explain that multivariable logistic regression was not feasible because only 10 positive events were observed, violating the rule of 10 events per variable and risking severe overfitting. Instead, we performed univariate Fisher’s exact tests and descriptive epidemiological summaries.

Diversity indices: We have calculated and added the Shannon diversity index (H′ = 0.45) and Simpson diversity index (0.28) for the ectoparasite community in the Results, reflecting the marked dominance of mites (83.3%) over ticks (16.7%). The calculation method has been added to the Methods.

GPS/ArcGIS:GPS data collection is described in the Methods (Sample Collection):”Geographic coordinates of each sampling site were recorded at the center of the pasture using mobile positioning software (accuracy ±5 m) and imported into ArcGIS 10.8 (ESRI, USA) for spatial analysis.”

**Comment 6:**The conclusion regarding vector-borne transmission risk is weakened by a discrepancy: the flea index is mentioned, but Table 4 lists only ticks and mites.

**Response:**This is a critical correction. We have removed all mention of “flea index”throughout the manuscript and replaced it with “ectoparasite index”(0.012). We have revised Table 6(formerly Table 4) to accurately reflect the identified taxa:hard ticks (Ixodidae) and mites (Acari). We explicitly state: “No fleas (Siphonaptera) were recovered in this survey.”

**Comment 7:**The limitations of the analysis are not clearly described.

**Response:**We have added a dedicated Limitations subsection in the Discussion, addressing five key constraints: (1) missing demographic data, which prevents host-level risk factor analysis; (2) cross-sectional design, which precludes inference of temporal dynamics; (3) low sensitivity of culture/PCR for transient carriers, which may yield false negatives; (4) non-significant spatial analysis, likely due to limited seropositive samples and broad geographic scale; and (5) absence of molecular barcoding for ectoparasites, which limits definitive taxonomic assignment.

**Comment 8:**The Discussion restates large portions of raw data from the Results.

**Response:**We have removed redundant numerical restatements from the Discussion. The revised Discussion focuses on ecological interpretation, public health implications, and comparison with Qinghai Province data.

**Comment 9:**Yersinia pestis should be italicised throughout, including Tables and Figure captions.

**Response:**We have checked the entire manuscript and ensured that Yersinia pestis and Y. pestis appear in italics in all sections, tables, and figure captions.

**Comment 10:**Results should be restructured into subsections; a spatial map should be included; Table 4 needs genus/species.

**Response:**

- Results are now divided into five subsections: (1) Sample Collection. (2) Serological Detection.(3)Spatial Distribution. (4) Ectoparasite Detection.(5)Molecular and Culture Results.

-Figure 1 (ArcGIS map) has been prepared showing altitudinal overlap and proximity to marmot foci.

- Table 6 (formerly Table 4) has been revised. we have added a footnote:“Note: Specimens were identified to family (Ixodidae) or subclass (Acari) level by morphological stereomicroscopy using standard taxonomic keys.”

**Reviewer #3**

**Comment 1:**The Abstract should prominently state the effort: 3,033 sheep, 35 herds, 9 villages.

**Response:**The Abstract has been revised. The first sentence of the Results now reads: “A total of 3,033 Tibetan sheep from 35 herds across 9 villages were sampled...”

**Comment 2**:A map visualising the different herds/villages and pasture geography would be a great addition.

**Response:**We have prepared Figure 1, an ArcGIS map showing village locations, herd sampling sites (positive vs. negative), and the geography of pastures relative to Himalayan marmot habitat zones.

**Comment 3:**Nasal swabs are used for PCR. Is that established? Why not blood PCR?

**Response:**Nasal swabs were selected as the primary specimen for active infection screening based on the national diagnostic standard WS279-2008, which recommends respiratory/lesion materials for pathogen isolation in live animals." Blood PCR was not performed because serological positivity indicates past exposure rather than active bacteremia. In surveillance of naturally occurring zoonotic diseases, nasal/respiratory specimens are preferred for detecting active infection in live animals, while blood is typically used for serological evidence of exposure.

**Comment 4:**Only 1,090 samples were PCR tested. Why not 3,033? A table showing which specimen was tested from which herd may help.

Response: We have added Table 4 (Distribution of Specimen Types by Village and Herd). “Due to resource constraints and the prioritization of pathogen detection in high-risk villages, nasal swabs were collected from 1,090 sheep (35.9% of total), while serum was collected from all 3,033 sheep to maximize serological coverage.”

**Comment 5:**Is the 0.3% prevalence the true rate, or do infected animals die and become removed? What does infection mean for sheep health?

**Response:**We clarify that 0.33% (10/3,033) is the seroprevalence of F1 antibodies, not the infection rate—it reflects historical exposure to Y. pestis rather than active infection. All 10 seropositive animals were clinically healthy at sampling, and no plague-related deaths were reported by herders or observed during the study period. However, we acknowledge two limitations:(1) Potential underestimation due to mortality: Our cross-sectional design cannot exclude the possibility that acutely infected sheep died and were removed from the population before sampling. This is a recognized challenge in prevalence studies of lethal pathogens.(2) Health impact: Available evidence from Qinghai Province suggests Tibetan sheep typically develop subclinical or mild infections with rapid pathogen clearance, consistent with their role as secondary/sentinel hosts rather than reservoir hosts. The low antibody titers (1:16–1:128) and absence of clinical signs in our seropositive animals support this pattern. Severe disease or death appears rare, though carcass handling poses zoonotic risk to humans.

**Comment 6:**Why are the few human cases described presumably caused by rodents or sheepdogs but not sheep? What is the implication for human health?

**Response:**As an even-toed ungulate (Artiodactyla), the Tibetan sheep exhibits significant differences from primary reservoir hosts such as rodents in terms of susceptibility to *Yersinia pestis* and the pathological course following infection. Current research indicates that interspecies responses to plague infection are highly species-specific: while some animals may manifest as asymptomatic carriers or transient bacteremic states, the immune system of Tibetan sheep likely possesses robust innate clearance capacity against *Y. pestis*. Upon infection, Tibetan sheep can rapidly mount specific adaptive immune responses, facilitating pathogen elimination within a relatively short timeframe, thereby precluding the establishment of persistent, high-level bacteremia.This biological characteristic accounts for the epidemiological observation in Qinghai Province, where only 14 *Y. pestis* strains were isolated from Tibetan sheep between 1975 and 2015 (mean isolation rate: approximately 0.35 strains/year). Despite the low bacterial carriage and isolation rate, serological surveillance of Tibetan sheep retains substantial public health monitoring value—Tibetan sheep can serve as”plague sentinel animals”, whose dynamic seroprevalence patterns effectively predict enzootic trends, providing scientific evidence for early warning and prevention strategies.

**Comment 7:**The introduction relies on a very low number of references.

**Response:**We have expanded the Introduction with additional foundational literature on Y. pestis ecology, Tibetan sheep epidemiology, and vector biology. We have also added the suggested citation regarding ancient Y. pestis lineages in sheep (see below).

Comment 8:Cite PMID: 40795857 on ancient Y. pestis lineage in sheep.

Response: We thank the reviewer for this excellent suggestion. We have added the following to the Background: "The role of sheep in plague ecology extends beyond contemporary transmission dynamics. A recent study identified a Bronze Age Y. pestis genome in a 4,000-year-old domesticated sheep from the Eurasian Steppe, belonging to the Late Neolithic Bronze Age (LNBA) lineage previously known only from ancient humans [10]. This lineage lacked the genetic toolkit for efficient flea-borne transmission, suggesting sheep served as important intermediate hosts in prehistoric plague spread. This deep evolutionary context underscores the public health significance of monitoring plague in modern pastoral systems." This citation strengthens the rationale for our study by connecting modern Tibetan sheep surveillance to millennia of sheep-plague co-evolution.
