# Supplementary material for: A study on Tibetan sheep in the Gansu plague-endemic area and the risk of plague transmission by their vectors
Source: PLoS Negl Trop Dis. 2026 Aug 14;20(8):e0014650. doi: 10.1371/journal.pntd.0014650 (PMC13492742; doi:10.1371/journal.pntd.0014650)
Supplement: Peer review history

# Peer review: Decision Letter 0

**DOI:** 10.1371/journal.pntd.0014650.r001

**Authors:** Vladimir L. Motin, Qu Cheng

19 May 2026

PNTD-D-26-00605

A Study on Tibetan Sheep in the Gansu Plague-Endemic Area and the Risk of Plague Transmission by Their Vectors

PLOS Neglected Tropical Diseases

Dear Dr. 安,

Thank you for submitting your manuscript to PLOS Neglected Tropical Diseases. After careful consideration, we feel that it has merit but does not fully meet PLOS Neglected Tropical Diseases's publication criteria as it currently stands. Therefore, we invite you to submit a revised version of the manuscript that addresses the points raised during the review process.

Please submit your revised manuscript within by Jul 18 2026 11:59PM. If you will need more time than this to complete your revisions, please reply to this message or contact the journal office at plosntds@plos.org. When you're ready to submit your revision, log on to https://www.editorialmanager.com/pntd/ and select the 'Submissions Needing Revision' folder to locate your manuscript file.

Please include the following items when submitting your revised manuscript:

* A letter that responds to each point raised by the editor and reviewer(s). You should upload this letter as a separate file labeled 'Response to Reviewers'. This file does not need to include responses to any formatting updates and technical items listed in the 'Journal Requirements' section below.

* A marked-up copy of your manuscript that highlights changes made to the original version. You should upload this as a separate file labeled 'Revised Manuscript with Track Changes'.

* An unmarked version of your revised paper without tracked changes. You should upload this as a separate file labeled 'Manuscript'.

If you would like to make changes to your financial disclosure, competing interests statement, or data availability statement, please make these updates within the submission form at the time of resubmission. Guidelines for resubmitting your figure files are available below the reviewer comments at the end of this letter.

As the corresponding author, your ORCID iD is verified in the submission system and will appear in the published article. PLOS supports the use of ORCID, and we encourage all coauthors to register for an ORCID iD and use it as well. Please encourage your coauthors to verify their ORCID iD within the submission system before final acceptance, as unverified ORCID iDs will not appear in the published article. Only the individual author can complete the verification step; PLOS staff cannot verify ORCID iDs on behalf of authors.

We look forward to receiving your revised manuscript.

Kind regards,

Vladimir L. Motin, Ph.D.

Academic Editor

PLOS Neglected Tropical Diseases

Qu Cheng

Section Editor

PLOS Neglected Tropical Diseases

Shaden Kamhawi

co-Editor-in-Chief

PLOS Neglected Tropical Diseases

orcid.org/0000-0003-4304-636XX

Paul Brindley

co-Editor-in-Chief

PLOS Neglected Tropical Diseases

orcid.org/0000-0003-1765-0002

**Journal Requirements:**

If the reviewer comments include a recommendation to cite specific previously published works, please review and evaluate these publications to determine whether they are relevant and should be cited. There is no requirement to cite these works unless the editor has indicated otherwise.

1) Please ensure that the CRediT author contributions listed for every co-author are completed accurately and in full.

At this stage, the following Authors/Authors require contributions: 文静 安, Wenjing An, Aiwei He, Guotian Ma, Hua Chun, geli aoer, Dingsheng Wang, Ruixun Gan, Guorui Wang, Jinxiao Xi, Hu Wang, Chengxin Zhang, Yanyan Huang, Bayier Caoge, Cunshou Zhao, and Daqin Xu. Please ensure that the full contributions of each author are acknowledged in the "Add/Edit/Remove Authors" section of our submission form.

The list of CRediT author contributions may be found here: https://journals.plos.org/plosntds/s/authorship#loc-author-contributions

2) Please provide an Author Summary. This should appear in your manuscript between the Abstract (if applicable) and the Introduction, and should be 150-200 words long. The aim should be to make your findings accessible to a wide audience that includes both scientists and non-scientists. Sample summaries can be found on our website under Submission Guidelines:

https://journals.plos.org/plosntds/s/submission-guidelines#loc-parts-of-a-submission

3) Thank you for including an Ethics Statement for your study. Please include:

i) The approval number(s), or a statement that approval was granted by the named board(s).

4) We note that your Data Availability Statement is currently as follows: "all raw data and analytical methods are fully documented and available for peer review and future research.". Please confirm at this time whether or not your submission contains all raw data required to replicate the results of your study. Authors must share the “minimal data set” for their submission. PLOS defines the minimal data set to consist of the data required to replicate all study findings reported in the article, as well as related metadata and methods (https://journals.plos.org/plosone/s/data-availability#loc-minimal-data-set-definition).

For example, authors should submit the following data:

1) The values behind the means, standard deviations and other measures reported;

2) The values used to build graphs;

3) The points extracted from images for analysis..

Authors do not need to submit their entire data set if only a portion of the data was used in the reported study.

If your submission does not contain these data, please either upload them as Supporting Information files or deposit them to a stable, public repository and provide us with the relevant URLs, DOIs, or accession numbers. For a list of recommended repositories, please see https://journals.plos.org/plosone/s/recommended-repositories.

If there are ethical or legal restrictions on sharing a de-identified data set, please explain them in detail (e.g., data contain potentially sensitive information, data are owned by a third-party organization, etc.) and who has imposed them (e.g., an ethics committee). Please also provide contact information for a data access committee, ethics committee, or other institutional body to which data requests may be sent. If data are owned by a third party, please indicate how others may request data access.

5) Please amend your detailed Financial Disclosure statement. This is published with the article. It must therefore be completed in full sentences and contain the exact wording you wish to be published.

1) State the initials, alongside each funding source, of each author to receive each grant. For example: "This work was supported by the National Institutes of Health (####### to AM; ###### to CJ) and the National Science Foundation (###### to AM)."

2) State what role the funders took in the study. If the funders had no role in your study, please state: "The funders had no role in study design, data collection and analysis, decision to publish, or preparation of the manuscript."

3) If any authors received a salary from any of your funders, please state which authors and which funders..

If you did not receive any funding for this study, please simply state: u201cThe authors received no specific funding for this work.u201d

**Reviewers' Comments:**

Reviewer's Responses to Questions

**Key Review Criteria Required for Acceptance?**

As you describe the new analyses required for acceptance, please consider the following:

**Methods**

-Are the objectives of the study clearly articulated with a clear testable hypothesis stated?

-Is the study design appropriate to address the stated objectives?

-Is the population clearly described and appropriate for the hypothesis being tested?

-Is the sample size sufficient to ensure adequate power to address the hypothesis being tested?

-Were correct statistical analysis used to support conclusions?

-Are there concerns about ethical or regulatory requirements being met?

Reviewer #1: 1. The objectives of the study are to evaluate the role of Tibetan sheep in plague transmission in Gansu Province. Specifically, the study stresses the risk of the introduction of Tibetan sheep into this province. To address this question the authors must provide more information about the trends in introduction and distribution of Tibetan sheep within the known plague endemic areas. The only available information is that “in recent years … a dramatic increase in Tibetan sheep populations.” Listing some locations where the sheep were investigated is not sufficient to design the study and address the stated objectives.

2. Despite the high number of sheep serologically tested, the absence of information regarding the geographical distribution of the samples and their proximity to the manifested plague makes the analysis less representative.

3. The total number of ectoparasites (30 samples obtained from >3,000 sheep) raises the question about collection technique. The procedure for collecting ectoparasites is absent. It could be challenging to collect fleas, sheep flies, and other ectoparasites from sheep. This question should be addressed.

4. It is essential to discuss and present the target gene that was used for molecular screening.

Reviewer #2: The objectives are generally articulated but they lack a clear testable hypothesis. The study aimed to characterise the epidemiological status of plague in Tibetan sheep and to evaluate transmission risk in Gansu Province. I noticed that specific biological expectations, such as the relationship between altitude and infection or the influence of marmot emergence on sheep flea profiles, are not stated. The introduction would benefit from a formal hypothesis, for example, predicting that the increased density of Tibetan sheep in marmot foci has led to a significant shift in the structure of the vector community.

The study design is a cross-sectional survey conducted in spring and autumn to align with marmot activity. Though this is appropriate for baseline prevalence data, the design is currently insufficient to address the vector exposure profiles mentioned in the objectives, as it lacks a longitudinal component to capture dynamic shifts in vector density. Also, there is insufficient methodological detail on how the herds were selected and whether the sampling was truly representative of the regional sheep population.

The population of Tibetan sheep is clearly defined and geographically relevant, given their role as significant secondary hosts in high-altitude plague foci. However, the description lacks essential demographic data, including the age, sex and reproductive status of the sampled individuals. These factors are critical for testing any epidemiological hypothesis, as host physiology and behaviour directly influence susceptibility to both plague infection and ectoparasite infestation.

The sample size of 3,033 Tibetan sheep is highly impressive and likely sufficient to provide adequate statistical power for a prevalence study. However, because the study lacks a formal power analysis or hypothesis testing for small effect sizes (such as the influence of environmental variables on low-titer seroprevalence), it is difficult to definitively confirm whether the sample size is optimised for the study's secondary objectives.

The statistical analysis currently used is too shallow to support high-level ecological or epidemiological conclusions. The authors relied on basic descriptive summaries and chi-square tests, which do not account for the data’s complexity. To fulfil the study objectives, the analysis should incorporate Logistic Regression or generalised linear models (GLMs) to identify risk predictors, as well as diversity indices such as Shannon or Simpson to quantify vector exposure profiles. Additionally, the mention of ArcGIS for spatial analysis is not supported by a corresponding methodology for GPS data collection in the field.

There are no major ethical concerns; the study appears to meet regulatory standards.

Reviewer #3: The methods are appropriate. An overview which sample underwent which methodology is requested in the review. (see also Summary and General Comments)

**********

**Results**

-Does the analysis presented match the analysis plan?

-Are the results clearly and completely presented?

-Are the figures (Tables, Images) of sufficient quality for clarity?

Reviewer #1: 1. Using the map, which displays sample sites and recent plague detections in marmots, may help to present the distribution of animals which have been identified as serologically positive.

2. Table 4 is entitled “Classification and Identification of Ectoparasites on Tibetan Sheep,” but lacks information about the taxonomic identification of arthropods. "Grassland Hard Tick" is hardly sufficient; similarly, "Chigger Mite."

3. The authors state the importance of the flea index as a key indicator of plague transmission risk when there are zero fleas obtained from sheep.

Reviewer #2: The analysis presented does not match the analysis plan described in the methodology. Although authors performed basic descriptive summaries and chi-square tests for categorical variables, mostly ecological and spatial objectives remain unaddressed. Also, there is no evidence of flea indices or diversity metrics being conducted to characterise the vector exposure profile mentioned in the introduction.

Results are presented descriptively but are incomplete in relation to the study’s stated goals. Vector profile data are inconsistent eg., mentioned report on flea index while identification results in Table 4 list only hard ticks and chigger mites with no fleas identified.

Figures and tables are mixed quality and need refinement for clarity. Example Table 4 is not clear it fails to include the flea discussed listing only ticks and mites. Also the methodology mentions spatial analysis in ArcGIS but when comes to results no maps or visual figures to clarify the high altitude overlap between marmot colonies and sheep grazing areas.

Reviewer #3: Yes, the analysis matches the research plan (see also Summary and General Comments)

**********

**Conclusions**

-Are the conclusions supported by the data presented?

-Are the limitations of analysis clearly described?

-Do the authors discuss how these data can be helpful to advance our understanding of the topic under study?

-Is public health relevance addressed?

Reviewer #1: Although the conclusion about switching plague transmission between marmots and Tibetan sheep sounds intriguing, it is not well supported by the data.

Reviewer #2: Conclusions are partially supported by the data presented.The conclusion regarding vector-borne transmission risk is weakened by a significant discrepancy in the parasite data. The authors conclude there is a risk from flea vectors, yet their identification table only lists ticks and mites leaving the flea index mentioned in the text unsupported by taxonomic evidence.

The limitations of the analysis are not clearly or sufficiently described in the manuscript. The authors briefly mention that a single-season cross-sectional survey may not fully reflect vector dynamics, but they fail to address more critical limitations. These include the lack of demographic data (age, sex) which could bias prevalence results, the potential for low sensitivity in the bacterial culture methods used, and the fact that the spatial analysis did not reach statistical significance, limiting the ability to define precise high-risk geographical zones.

The authors discuss how these data advance understanding of plague ecology, specifically by highlighting the quiescence paradox, in which sheep show exposure despite the absence of active outbreaks. They suggest that Tibetan sheep serve as sentinel animals, providing serological evidence of plague activity within the Himalayan marmot foci. However, this point is not clear because the authors do not provide a detailed ecological profile of the vectors involved, which is necessary to understand the specific mechanisms of interspecific pathogen transmission.

Public health relevance is addressed, primarily through the recommendation to include Tibetan sheep in the national plague surveillance system. The authors correctly identify a complete marmot-Tibetan sheep-human transmission chain and advocate for health education and behavioral interventions for high-risk groups like herders and slaughterhouse workers.

Reviewer #3: The conclusions are well presented. (see also Summary and General Comments)

**********

**Editorial and Data Presentation Modifications?**

Use this section for editorial suggestions as well as relatively minor modifications of existing data that would enhance clarity. If the only modifications needed are minor and/or editorial, you may wish to recommend “Minor Revision” or “Accept”.

Reviewer #1: The limitations of the provide analysis should be addressed.

Reviewer #2: Based on the inconsistencies observed between the methodology, results and discussion, the following editorial and data presentation modifications are required to ensure scientific accuracy and professional clarity.

A major editorial correction is required regarding the Flea Index mentioned in the discussion, while results shows flea index of 0.012, Table 4 lists only Hard Ticks and Chigger Mites as the identified ectoparasites. The authors must resolve this discrepancy either by adding the identified flea species to the table or by revising the index terminology to a general ectoparasite index. Also, make sure that Yersinia pestis is italicised throughout the manuscript, including within the Tables and Figure captions.

Results section currently relies on dense and descriptive paragraphs. These should be restructured into subsections to improve readability. Also, authors should include a spatial map generated from their ArcGIS analysis to visualize the altitudinal overlap between Tibetan sheep grazing sites and Himalayan marmot habitats. Refinement in Table 4 is needed to be more specific. If Taxonomic Identification was performed as stated in the methodology, the genus and species of the grass ticks and chigger mites should be listed rather than broad categories.

Discussion section currently restates large portions of raw data from the results. These should be removed and replaced with interpretive text that focuses on the ecological implications of these findings.

Reviewer #3: Only very few references are used, which I am not familiar with when publishing on the Y. pestis topic. (see also Summary and General Comments)

**********

**Summary and General Comments**

Use this section to provide overall comments, discuss strengths/weaknesses of the study, novelty, significance, general execution and scholarship. You may also include additional comments for the author, including concerns about dual publication, research ethics, or publication ethics. If requesting major revision, please articulate the new experiments that are needed.

Reviewer #1: Despite the limited number of positive findings, the study is still important and interesting given the potential risk associated with the introduction of Tibetan sheep in plague-endemic territory within Gansu. The importance of the study is highlighted by the recommendations for including testing of Tibetan sheep in the regional plague surveillance system.

Reviewer #2: This manuscript presents a serological survey of Yersinia pestis in Tibetan sheep within the Himalayan marmot plague foci of Gansu Province. The study’s primary strength lies in its extensive sampling effort involving over 3,000 animals, which provides a high-powered snapshot of plague exposure in a region where sheep have been recently introduced for production. The finding of a 3.3‰ seropositivity rate is significant as it confirms that Tibetan sheep are integrated into the local ecological cycle of plague, likely serving as sentinel animals that provide a serological record of environmental pathogen activity.

However, the manuscript's overall require substantial improvement to meet international publication standards. Although the serological data are strong, the ecological characterisation of vector exposure profiles remains incomplete and inconsistent. There is a critical disconnect between the identification results and the discussion, and the statistical analysis does not fully use the large dataset provided.

Major weaknesses and reeded Revisions

•A major revision is required to resolve the discrepancy regarding the flea index. The text calculates a flea index of 0.012, yet the classification tables only list ticks and mites. For the vector profile objective to be considered met, the authors must provide specific taxonomic identification (genus and/or species) for all collected parasites, especially if fleas were indeed recovered.

•Current analysis is purely descriptive. Given the scale of the study, the authors should move beyond simple frequency tests

•To support the vector profile objective, the authors should apply biodiversity indices such as Shannon or Simpson indices to describe the ectoparasite community structure. This would add evidence to their claims of host-switching between marmots and sheep.

•The authors should provide a spatial map showing the proximity of positive sheep herds to known marmot plague foci. This visualisation is critical for readers to understand the geographical risk and the niche overlap argued in the discussion.

Novelty and Significance

The novelty of this work lies in identifying Tibetan sheep as potential sentinel animals in the Gansu foci. This distinction is crucial as it suggests that while sheep may not maintain the plague focus themselves (acting as terminal hosts), they are highly effective biological indicators for surveillance. If the authors can strengthen the link between the sheep's serological status and the specific vector species found on them, this study will make a meaningful contribution to understanding cross-species plague transmission on the Qinghai-Tibet Plateau.

Ethics and Publication Standards

There are no major concerns regarding research ethics, as the sampling was non-lethal and conducted with appropriate institutional approval. However, the authors must ensure that the bibliography is updated to include citations for the foundational ecological concepts mentioned in the introduction.

Although the data are valuable, a Major Revision is necessary to refine the analysis, resolve inconsistencies in vector data and improve the overall interpretive quality of the discussion.

Reviewer #3: Wenjing An et al present an investigation testing over 3000 Tibetan sheep from 35 herds across 9 villages to test for evidence of past or present Y. pestis infection. They collect and analyse venous blood, nasal swabs, and external parasites were collected from Tibetan sheep within the study area. Y. Pestis is the causative agent of plague - a major contributor to human disease outbreaks in the past. The investigation at hand finds that overall a low number of sheep (0.3%) show seroprevalence , and no evidence for an active infection.

The study provides important information on the current epidemiology of Y. pestis in one of its last endemic areas. The public health threat of Y. pestis is considerable given its high virulence and as such the study presents important insights into enzootic potential of Y. pestis in China. I have only a few comments and recommend publication with minor revisions.

- the abstract should state the effort that has been made. Namely 3033 sheep, 35 herds, 9 villages. Currently it is only implied, but it deserves more prominent placement in my opinion.

- a map visualising the different herds/ villages and ideally the pastures geography would be a great add. It could guide the interpretation whether or not plague foci are connected.

- Nasal swabs are used to detect the Y. pestis pathogen using PCR. Is that established? I understand the blood samples were all culture negative but a PCR on blood could have been done, too? Why not? That may provide additional evidence for the conclusion that no active infection is happening.

- Only 1090 samples were PCR tested. Why not 3033? A table that shows which type of specimen was tested from which herd/ village may help to assess the PCR-based findings

- I am wondering about the 0.3% prevalence of Y. pestis positive antibodies. Is that the true rate, or do infected animals die and become removed from the population? What does it mean for the sheep’s health to be infected with Y. pestis? Anything known?

- Why are the few human cases described presumably caused by rodents or sheepdog sources but not sheep? Is there any reason to believe that dogs carry active infections (life pathogens) longer than sheep? Or is there simply more interaction between humans and dogs which thus enables transmission? What is ultimately the implication of Y. pestis infection in sheep for human health?

- More generally, the introduction relies on a very low number of references. Are those few encompassing the breadth of research on Y. pestis?

- A recent paper (PMID: 40795857) identified an ancient Y. pestis lineage in sheep - a lineage otherwise only found in ancient humans and without the genetic toolkit to be efficiently transmitted by fleas - arguing that sheep had been important intermediate hosts in the human past. This work highlights the importance of sheep in terms of human public health going back 4000 years and may help build the case for this study in the introduction.

**********

PLOS authors have the option to publish the peer review history of their article (what does this mean?). If published, this will include your full peer review and any attached files.

If you choose “no”, your identity will remain anonymous but your review may still be made public.

**Do you want your identity to be public for this peer review?**  For information about this choice, including consent withdrawal, please see our Privacy Policy.

Reviewer #1: No

Reviewer #2: No

Reviewer #3: No

[NOTE: If reviewer comments were submitted as an attachment file, they will be attached to this email and accessible via the submission site. Please log into your account, locate the manuscript record, and check for the action link "View Attachments". If this link does not appear, there are no attachment files.]

**Figure resubmission:**

While revising your submission, we strongly recommend that you use PLOS’s NAAS tool (https://ngplosjournals.pagemajik.ai/artanalysis) to test your figure files. NAAS can convert your figure files to the TIFF file type and meet basic requirements (such as print size, resolution), or provide you with a report on issues that do not meet our requirements and that NAAS cannot fix.

After uploading your figures to PLOS’s NAAS tool - https://ngplosjournals.pagemajik.ai/artanalysis, NAAS will process the files provided and display the results in the "Uploaded Files" section of the page as the processing is complete. If the uploaded figures meet our requirements (or NAAS is able to fix the files to meet our requirements), the figure will be marked as "fixed" above. If NAAS is unable to fix the files, a red "failed" label will appear above. When NAAS has confirmed that the figure files meet our requirements, please download the file via the download option, and include these NAAS processed figure files when submitting your revised manuscript.

**Reproducibility:**

To enhance the reproducibility of your results, we recommend that authors of applicable studies deposit laboratory protocols in protocols.io, where a protocol can be assigned its own identifier (DOI) such that it can be cited independently in the future. Additionally, PLOS ONE offers an option to publish peer-reviewed clinical study protocols. Read more information on sharing protocols at https://plos.org/protocols?utm_medium=editorial-email&utm_source=authorletters&utm_campaign=protocols

Attachment Submitted filename: PNTD-D-26-00605_reviewer_edits.pdf

# Author comment: Author response to Decision Letter 1

**DOI:** 10.1371/journal.pntd.0014650.r002

7 Jul 2026

Attachment Submitted filename: Response to Reviewers.docx

# Peer review: Decision Letter 1

**DOI:** 10.1371/journal.pntd.0014650.r003

**Authors:** Vladimir L. Motin, Qu Cheng

5 Aug 2026

Dear Daqin Xu xu,

We are pleased to inform you that your manuscript 'A Study on Tibetan Sheep in the Gansu Plague-Endemic Area and the Risk of Plague Transmission by Their Vectors' has been provisionally accepted for publication in PLOS Neglected Tropical Diseases.

Before your manuscript can be formally accepted you will need to complete some formatting changes, which you will receive in a follow up email. A member of our team will be in touch with a set of requests.

Please note that your manuscript will not be scheduled for publication until you have made the required changes, so a swift response is appreciated.

IMPORTANT: The editorial review process is now complete. PLOS will only permit corrections to spelling, formatting or significant scientific errors from this point onwards. Requests for major changes, or any which affect the scientific understanding of your work, will cause delays to the publication date of your manuscript.

Should you, your institution's press office or the journal office choose to press release your paper, you will automatically be opted out of early publication. We ask that you notify us now if you or your institution is planning to press release the article. All press must be co-ordinated with PLOS.

Thank you again for supporting Open Access publishing; we are looking forward to publishing your work in PLOS Neglected Tropical Diseases.

Best regards,

Vladimir L. Motin, Ph.D.

Academic Editor

PLOS Neglected Tropical Diseases

Qu Cheng

Section Editor

PLOS Neglected Tropical Diseases

Shaden Kamhawi

co-Editor-in-Chief

PLOS Neglected Tropical Diseases

orcid.org/0000-0003-4304-636XX

Paul Brindley

co-Editor-in-Chief

PLOS Neglected Tropical Diseases

orcid.org/0000-0003-1765-0002

***********************************************************

all reviewers found the author's response satisfactory

Reviewer's Responses to Questions

**Key Review Criteria Required for Acceptance?**

As you describe the new analyses required for acceptance, please consider the following:

**Methods**

-Are the objectives of the study clearly articulated with a clear testable hypothesis stated?

-Is the study design appropriate to address the stated objectives?

-Is the population clearly described and appropriate for the hypothesis being tested?

-Is the sample size sufficient to ensure adequate power to address the hypothesis being tested?

-Were correct statistical analysis used to support conclusions?

-Are there concerns about ethical or regulatory requirements being met?

Reviewer #1: No more questions.

Reviewer #2: The revised manuscript has addressed the methodological concerns raised previously. The study objectives are now clearly stated as three focused research questions that suit the cross-sectional design and herd selection is explained. Ethics approval is confirmed, sample size is well justified and the statistical approach is appropriate given the small number of positive cases.

Reviewer #3: Following revision the study design is well presented and the methodology is appropriate.

**********

**Results**

-Does the analysis presented match the analysis plan?

-Are the results clearly and completely presented?

-Are the figures (Tables, Images) of sufficient quality for clarity?

Reviewer #1: No more questions.

Reviewer #2: The results are clearly structured into five subsections and match the described analysis plan.Sample collection numbers are consistently reported and a map has been added showing the geographic distribution of sampling sites relative to plague detection sites.

Reviewer #3: All three yes.

**********

**Conclusions**

-Are the conclusions supported by the data presented?

-Are the limitations of analysis clearly described?

-Do the authors discuss how these data can be helpful to advance our understanding of the topic under study?

-Is public health relevance addressed?

Reviewer #1: No more questions.

Reviewer #2: The conclusions are broadly supported by the data and the public health relevance is well articulated, particularly the recommendation to include Tibetan sheep in routine plague surveillance and the identification of a complete marmot-sheep-human transmission chain. Limitations are clearly described in a dedicated subsection covering five key constraints. However, the conclusion that detection of Ixodidae and Acari confirms vector-host switching between marmots and Tibetan sheep is stronger than the evidence supports, since not all members of these groups are confirmed plague vectors.

The authors discuss the implications of their findings well in terms of advancing understanding of plague ecology in pastoral systems and informing prevention strategies for high-risk populations.

Reviewer #3: The conclusions are clear and the limitations are now explained in more detail. The importance of the study is explained.

**********

**Editorial and Data Presentation Modifications?**

Use this section for editorial suggestions as well as relatively minor modifications of existing data that would enhance clarity. If the only modifications needed are minor and/or editorial, you may wish to recommend “Minor Revision” or “Accept”.

Reviewer #1: Multiple orthographic mistakes, e.g. brakes between words, appeared in the revised version.

Reviewer #2: This manuscript is accepted with minor revisions. The only remaining change is in the Discussion. The paragraph on ectoparasites should begin with an ecological interpretation rather than repeating the numerical results. Also the statement about vector–host switching should be reframed to reflect that identification was at family level only and that species-level molecular confirmation would be needed to fully support that conclusion.

Reviewer #3: Accept

**********

**Summary and General Comments**

Use this section to provide overall comments, discuss strengths/weaknesses of the study, novelty, significance, general execution and scholarship. You may also include additional comments for the author, including concerns about dual publication, research ethics, or publication ethics. If requesting major revision, please articulate the new experiments that are needed.

Reviewer #1: The authors have seriously taken all my previous comments.

Reviewer #2: (No Response)

Reviewer #3: I appreciate the revisions done by An et al. In particular, the new Figure 1 (map) and additional tables improved the manuscript, which I consider suitable for publication.

**********

PLOS authors have the option to publish the peer review history of their article (what does this mean?). If published, this will include your full peer review and any attached files.

If you choose “no”, your identity will remain anonymous but your review may still be made public.

**Do you want your identity to be public for this peer review?**  For information about this choice, including consent withdrawal, please see our Privacy Policy.

Reviewer #1: No

Reviewer #2: No

Reviewer #3: No

Attachment Submitted filename: PNTD-D-26-00605_R2_edits.pdf

# Editor report: Acceptance letter

**DOI:** 10.1371/journal.pntd.0014650.r004

**Authors:** Vladimir L. Motin, Qu Cheng

Dear Daqin Xu xu,

We are delighted to inform you that your manuscript, "A Study on Tibetan Sheep in the Gansu Plague-Endemic Area and the Risk of Plague Transmission by Their Vectors," has been formally accepted for publication in PLOS Neglected Tropical Diseases.

We have now passed your article onto the PLOS Production Department who will complete the rest of the publication process. All authors will receive a confirmation email upon publication.

The corresponding author will soon be receiving a typeset proof for review, to ensure errors have not been introduced during production. Please review the PDF proof of your manuscript carefully, as this is the last chance to correct any scientific or type-setting errors. Please note that major changes, or those which affect the scientific understanding of the work, will likely cause delays to the publication date of your manuscript. Note: Proofs for Front Matter articles (Editorial, Viewpoint, Symposium, Review, etc...) are generated on a different schedule and may not be made available as quickly.

Soon after your final files are uploaded, the early version of your manuscript will be published online unless you opted out of this process. The date of the early version will be your article's publication date. The final article will be published to the same URL, and all versions of the paper will be accessible to readers.

For Research Articles, you will receive an invoice from PLOS for your publication fee after your manuscript has reached the completed accept phase. If you receive an email requesting payment before acceptance or for any other service, this may be a phishing scheme. Learn how to identify phishing emails and protect your accounts at https://explore.plos.org/phishing.

Thank you again for supporting open-access publishing; we are looking forward to publishing your work in PLOS Neglected Tropical Diseases.

Best regards,

Shaden Kamhawi

co-Editor-in-Chief

PLOS Neglected Tropical Diseases

Paul Brindley

co-Editor-in-Chief

PLOS Neglected Tropical Diseases
